# On the Identifiability of 3- and 4-Parameter Item Response Theory Models From the Perspective of Knowledge Space Theory

**DOI:** 10.1007/s11336-024-09950-z

**Published:** 2024-02-13

**Authors:** Stefano Noventa, Sangbeak Ye, Augustin Kelava, Andrea Spoto

**Affiliations:** 1 Methods Center, Universität Tübingen, Tübingen, Germany; 2 Department of General Psychology, University of Padova, Padua, Italy

**Keywords:** knowledge space theory, item response theory, identifiability, empirical indistinguishability, rasch models, 3PL, 4PL

## Abstract

The present work aims at showing that the identification problems (here meant as both issues of empirical indistinguishability and unidentifiability) of some item response theory models are related to the notion of identifiability in knowledge space theory. Specifically, that the identification problems of the 3- and 4-parameter models are related to the more general issues of forward- and backward-gradedness in all items of the power set, which is the knowledge structure associated with IRT models under the assumption of local independence. As a consequence, the identifiability problem of a 4-parameter model is split into two parts: a first one, which is the result of a trade-off between the left-side added parameters and the remainder of the Item Response Function, e.g., a 2-parameter model, and a second one, which is the already well-known identifiability issue of the 2-parameter model itself. Application of the results to the logistic case appears to provide both a confirmation and a generalization of the current findings in the literature for both fixed- and random-effects IRT logistic models.

## Introduction

A statistical model is identifiable if no two different sets of parameters values are observationally equivalent, i.e., they yield the same likelihood of the observed outcomes (see, e.g., Bamber and Van Santen, 2000; McCullagh, 2002, for a detailed introduction to the topic). If identifiability cannot be established, the parameters of the model can neither be interpreted in a meaningful way nor be consistently estimated unless additional restrictions are imposed. Unidentifiability that occurs within (outside) the neighborhood of a given point in the parameter space is said to be local (global). While local unidentifiability implies an infinity of equivalent reparameterizations and bears strong theoretical relevance but can always be solved by setting additional restrictions, global unidentifiability has a more practical relevance as it is associated with global indeterminacy issues and might require ad hoc methods to be handled. The present manuscript focuses on issues of local identifiability in item response theory (IRT) and knowledge space theory (KST), but it does so within a more general form of the identification problem. Indeed, identification issues in latent variable models might occur not only in relation to an observationally equivalent set of parameters but also in the presence of specification problems that concern the mathematical forms of both the distribution of the latent variables and/or the relationships between observed and latent variables (Koopmans and Reiersøl, 1950). Following the terminology used by Ip (2010), we consider the situation in which different models are observationally equivalent, i.e., they possess different mathematical forms of the distribution of the latent variables and/or of the relationships between observed and latent variables but yield the same likelihood of the observed outcomes, an issue of ‘empirical indistinguishability’ rather than unidentifiability. In spite of their relevance, identification problems in IRT models are still a topic under investigation. In the present work, our aim is to fit another piece to the puzzle by showing that the identification problems of some IRT models can be grounded in the notion of identifiability in KST.

Although by construction, the KST framework does not account for latent traits, it can be extended to a full IRT approach (Noventa et al., 2019). The resulting KST-IRT framework encompasses most IRT models and, under the necessary condition that the knowledge structure taken under consideration is the power set of the items so that local independence is captured, it establishes the equivalence of guessing and slipping parameters in 4-parameter IRT models with, respectively, lucky guesses and careless error parameters in the KST Basic Local Independence Model (BLIM; e.g., Doignon and Falmagne, 1999; Falmagne and Doignon, 1998). Following the terminology of Thissen and Steinberg (1986), we refer to these error parameters as left-side added parameters. By relying on a KST-IRT approach, in the present work, it is shown that the identification issues of the 3- and 4-parameter IRT models are related to a more general issue of identifiability arising in knowledge structures in presence of forward- or backward- gradedness w.r.t. an item (i.e., by, respectively, adding or removing an item from a knowledge state of a knowledge structure one still obtains a state of the structure). As the knowledge structure associated with the requirement of local stochastic independence in IRT is the power set, such structure is both forward- and backward-graded in all of the items, and as a result, one has a trade-off between the left-side added parameters and the knowledge state probabilities for all items. The KST identifiability problem translates, in the KST-IRT case, into an issue of both identifiability and empirical indistinguishability involving a trade-off between the left-sided added parameters and the parameters within the remainder of an Item Response Function (IRF), e.g., the Rasch model or the 2-Parameter Logistic model. This splits the identification problem into two parts: first, the identification issue following from the trade-off between the left-sided added parameters and the IRF to which such parameters are added; second, the identifiability issue concerning the latter IRF. The issue is one of both identifiability and empirical indistinguishability in the following sense: If the KST transformations are applied to the IRT case, they provide alternative IRFs that are observationally equivalent to the initial ones. This is an issue of empirical indistinguishability as different models yield the same distribution of the outcomes. If the KST transformations are instead applied to the IRT case while also assuming that the mathematical form of the IRFs must be held constant, then they provide alternative reparameterizations of the IRFs (local identifiability issue of the IRFs) that, however, require different distributions of the latent variables (empirical indistinguishability issue) and thus actually correspond to different statistical models, with possibly different substantive assumptions and inferences. After a brief introduction of the identifiability issue in IRT, KST notions are introduced, and then, the link between the two approaches is established. General transformations for 3- and 4-parameter IRT models are provided in light of the general frameworks. Their specific application to the logistic function is then discussed as a sub-case.

## Identifiability in IRT

### General Notions

The Rasch model (RM), also known as 1-Parameter Logistic (1PL) model, is nested within the 2-Parameter Logistic (2PL) model, which is in turn nested within the 3-Parameter Logistic (3PL) and 4-Parameter Logistic (4PL) models. The form of the latter is here given by:

1 $$\begin{aligned} P(X_i = 1|\theta , \Gamma ^4_i)&= c_i+ (1-d_i -c_i)\frac{e^{a_i(\theta -b_i)}}{1+e^{a_i(\theta -b_i)}} = \frac{(1-d_i)e^{a_i\theta }+c_ie^{a_ib_i}}{e^{a_i\theta }+e^{a_ib_i}} \end{aligned}$$

where $\Gamma ^4_i=\{b_i,a_i,c_i,d_i\}$ contains the difficulty parameter $b_i$ of the RM, the discrimination parameter $a_i$ of the 2PL, the guessing parameter $c_i$ of the 3PL (Birnbaum, 1968), and the slipping parameter $d_i$ of the 4PL (Barton and Lord, 1981). A common formulation of the IRF (1) replaces $1-d_i$ with $d_i$ so that $d_i$ captures the upper asymptote of the IRF. In the present work, since we are interested in relating KST and IRT left-side added parameters, the notation of Equation (1) is more convenient.

Equation (1) is written using a random-effects notation, in which the ability is treated as a latent variable. If instead a fixed-effects notation is considered, abilities are incidental parameters $\theta _j$ for $j \in \{1,\ldots , N\}$, with *N* the number of persons. The IRF (1) can be given notation $P(X_{ji} = 1|\theta _j, \Gamma ^n_i)$ with $X_{ji}$ the response of the *j*-th individual to the *i*-th item. Random- and fixed-effects perspectives are, respectively, grounded within a ‘random sampling’ view and a ‘stochastic subject’ view of the IRT process (see, e.g., Holland, 1990). In the former, an IRF like (1) represents the proportion of individuals with a level of ability $\theta$ that provides a correct answer to the *i*-th item. In the latter, the IRF represents the probability of the *j*-th individual to answer correctly the *i*-th item. Typical estimation methods for the fixed-effects case are joint maximum likelihood (JML) and conditional maximum likelihood (CML). CML is, however, restricted to the Rasch-family of models, while JML is well known to provide inconsistent estimates of $b_i$ due to the incidental parameters problem (see, e.g., Haberman, 1977; Andersen, 1980; Ghosh, 1995). For these reasons, the fixed-effects perspective is often used for didactic purposes, while a random-effect perspective is preferred in practice. With a random-effect specification, ability can be integrated out when using marginal maximum likelihood (MML). A final remark on the $\Gamma ^n_i$ notation, in which *n* is the number of item parameters. The RM or 1PL model is associated with $\Gamma ^1_i=\{b_i\}$, the 2PL model to $\Gamma ^2_i=\{b_i,a_i\}$, the 3PL model to $\Gamma ^3_i=\{b_i,a_i,c_i\}$, and the 4PL model to $\Gamma ^4_i=\{b_i,a_i,c_i,d_i\}$. $\Gamma ^{1,3}_i=\{b_i,c_i\}$ is used for the 1-Parameter model plus Guessing (1PL-G), $\Gamma ^{1,4}_i=\{b_i,d_i\}$ is used for the 1-Parameter model plus Slipping (1PL-S), and $\Gamma ^{-2}_i=\{b_i,c_i,d_i\}$ is used for the 4PL without discrimination, which is labeled as 1PL-GS.

### Unidentifiability of IRT Models

It is well known that the parameters of the RM are not unique since a uniform translation of both ability $\theta$ and difficulty $b_i$ for the same constant yields the same response probability. This is the only form of local unidentifiability for the RM/1PL. Additionally, in the 2PL model, one can dilate person and difficulty parameters by the same constant while dilating the discrimination parameter $a_i$ by the reciprocal. As to the guessing parameter $c_i$, it has been a source of debate in the literature. Some problematic features pertaining the global unidentifiability of the 3PL model had already been discussed by Samejima (1973), i.e., the nonuniqueness of the maximum for the likelihood w.r.t. the latent trait $\theta$ (see also Yen et al., 1991). Instability of the estimates has also been long known in the literature. Thissen and Wainer (1982) highlighted that estimation of the *c*-parameters can “wreak havoc” with the estimation of the *b*-parameters since easy items have few observations low enough to provide enough information. A similar problem can occur for very difficult items (see, e.g., Drasgow and Parsons, 1983; Hulin et al., 1982). Instability also does not concern only the difficulty parameters; van Der Linden and Hambleton (1997) stated that small changes in the guessing parameter can be compensated by small changes in the slope of the curve. More in general, Mislevy (1986) highlighted that the instability of the ML estimates in the 3PL model stems from the fact that different triples $\Gamma _i^3$ “can trace similar IRFs in the region of the ability scale where the sample of examinees is to be found” often resulting in nearly flat likelihood surfaces. Trade-offs seem to occur between *c* and all the other parameters. Lack of stability of the estimates of the 3PL model has also been discussed by Patz and Junker (1999), DeMars (2001), and Pelton (2002). As a consequence of the difficulty in estimating the lower asymptote of the 3PL, the 4PL model is often considered even more problematic to estimate (see, e.g., Embretson and Reise, 2000; Baker and Kim, 2004). Nonetheless, it is worth mentioning that there has recently been a renewed interest in 4-Parameter models (see, e.g., Hessen, 2005; Loken and Rulison, 2010; Ogasawara, 2012; Culpepper, 2016).

Local identifiability of IRT models is typically assessed by establishing an injective mapping between the parameters of interest of the model and the identified parametrization associated with the experimental outcomes. The choice of both parameters of interest and identified parametrization depends on whether the model is given a fixed-effects, a random-effects, or a semi-parametric specification (see, e.g., San Martín et al., 2009; San Martín and Rolin, 2013; San Martín, 2016). Interpretation of the parameters based on the identification analysis was provided by Fariña et al. (2019). In the fixed-effects case, the parameters of interest consist of the item parameters in $\Gamma _i^n$ and the latent abilities $\theta _j$ for $j \in \{1,\ldots , N\}$, while the identified parametrization is given by the parameters of mutually independent Bernoulli distributions. In the random-effects case, the parameters of interest are the item parameters in $\Gamma _i^n$ and the scale $\sigma$ and location $\mu$ parameters of some distribution $f(\theta ; \mu , \sigma )$ of the individual’s ability, while the identified parametrization is given by the parameters of a Multinomial distribution associated with the response patterns. Finally, the semi-parametric case follows the same reasoning as the random-effects case, but the distribution of the ability itself is treated as a parameter. In what follows, we are interested in the results for fixed- and random-effects models.

Although the 3PL model was initially believed to be always identifiable (see, e.g., Lord, 1980), Maris (2002) and Maris and Bechger (2009) showed that trade-offs can occur between the parameters of the 1PL-G model in a non-trivial way. Indeed, the model of Eq. (1), in the case $d_i=0$ and when all discrimination parameters are equal (i.e., 1PL-G model, $a_i=1$), is unidentifiable since the transformations

2 $$\begin{aligned} {\left\{ \begin{array}{ll} c_i^* = \frac{c_ie^{b_i}-\ell }{e^{b_i}-\ell }\\ e^{\theta ^*}= e^{\theta }+\ell \\ e^{b_i^*}= e^{b_i}-\ell \end{array}\right. } \end{aligned}$$

yield equivalent response probabilities

$$\begin{aligned} P(X_i=1|\theta ^*, \Gamma ^{1,3*}_i)&= \frac{e^{\theta ^*}+c_i^*e^{b_i^*}}{e^{\theta ^*}+e^{b_i^*}} = \frac{e^{\theta }+\ell +c_ie^{b_i}-\ell }{e^{\theta }+l+e^{b_i}-\ell } = \frac{e^{\theta }+c_ie^{b_i}}{e^{\theta }+e^{b_i}} = P(X_i=1|\theta , \Gamma ^{1,3}_i). \end{aligned}$$

Notice that by transformations (2) it follows that $\ell \in [-e^{\min {\theta }},e^{\min _i{b_i}})$. If a fixed-effects interpretation of $\theta$ is considered, there is a minimal value $\theta _m = \min _{j}{\theta _j}$ such that $e^{\min {\theta }}=e^{\theta _m}$. If instead a random-effects interpretation is considered, one has that $\displaystyle \lim _{\theta \rightarrow -\infty } e^{\min {\theta }}=0$ so that $\ell \in [0,e^{\min _i{b_i}})$. It should be stressed that transformations (2) provide an unidentifiability result in the fixed-effects case, but in the random-effects case they are an example of empirically indistinguishable statistical models. Indeed, in spite of the fact that the mathematical forms of the IRFs stay the same, the mathematical form of the distribution of the latent variable changes under the transformations. As a matter of fact, the second part of the work of Maris and Bechger (2009) provides an example of empirically indistinguishable alternative models.

As the results of Maris and Bechger (2009) cast doubts on the identifiability of the 3PL model, or of at least some degenerate case of it, several authors investigated the local identifiability of both fixed- and random-effects IRT models. A general discussion about identifiability conditions for 1PL, 2PL, and 1PL-G models for both fixed- and random-effects models can be found in San Martín (2016). Results for the random-effects 1PL, 2PL, and 1PL-G models have been discussed by San Martín and Rolin (2013), San Martín et al. (2013). Specifically, the parameters $b_i$, $c_i$, $\mu$, and $\sigma$ of the random-effects 1PL-G model are identified if (1) at least three items are available, (2) the guessing parameter $c_1$ is set to zero, and (3) the traditional linear restrictions (e.g., $b_1=0$ or $\sum _ib_i =0$ ) are imposed to remove the unidentifiability of the 1PL model. Up to our knowledge, there are no results for the random-effects 3PL and 4PL models.

More results are instead available for the fixed-effects models. van der Linden and Barrett (2016) showed that identifiability issues arise also in the degenerate case in which all individuals have the same ability values. It has been shown that the fixed-effects 1PL-G model is identifiable if difficulty and guessing parameters of one item are set to known constants, as in the random-effects case, and at least two persons with different abilities are available (San Martín et al., 2013). An alternative set of restrictions (two items with equal guessing but distinct difficulties) was later provided by Ogasawara (2020). San Martín et al. (2015) argued that the 3PL is unidentified, but this latter result was rectified by Wu (2016), which showed that as long as four persons with distinct abilities and two items with distinct discrimination parameters are available, the fixed-effects 3PL model is identifiable up to the usual permissible transformations of the 2PL model. These sufficient conditions were also shown to be necessary by Ogasawara (2017; 2020). Hence, in the case of the fixed-effects 3PL model, the belief of the locally identified 3PL model as found in Lord (1980) is still generally correct in that both the cases considered in Maris and Bechger (2009) and van der Linden and Barrett (2016) are special degenerate cases of the 3PL model with added restrictions to their parameters. As Wu (2016) pointed out, although it is not unusual for the parameter space of an identified model to contain sub-spaces in which the model is unidentified, these situations are still relevant since they might impact procedures like likelihood-ratio testing. Cases of identifiability and unidentifiability for the fixed-effects 3PL (and 4PL) were also recently summarized, discussed, and extended by Ogasawara (2017; 2020). More in detail, Ogasawara (2017) systematized the previous results and provided a wider set of unidentified models, extended the analysis to non-logistic fixed-effects 3-parameter models by briefly discussing the probit model, and by exploring the unidentifiability of the general family of IRFs of the form

3 $$\begin{aligned} P(X_i = 1|\theta , \Gamma _i^{1,3}) = c_i+(1-c_i)\frac{G(\theta )}{G(\theta )+H(b_i)} \end{aligned}$$

for arbitrary strictly increasing functions *G* and *H*. Ogasawara (2017, Proposition 2) showed that the 4PL is identified under the same conditions of Wu (2016) plus either the $c_i$’s or the $d_i$’s are given and that in the 1PL-GS model there exist further trade-offs of parameters in addition to those highlighted by transformations (2). Specifically, the following transformations of the left-side added parameters

4 $$\begin{aligned} {\left\{ \begin{array}{ll} (1-d_i^*) = (1-d_i) + k^*\\ c_i^* = c_i - \frac{k^*P(X_{i,j}=1|\theta _j, \Gamma ^{1}_i)}{1-P(X_i=1|\theta , \Gamma ^{1}_i)} = c_i- k^*e^{\theta _j-b_i} \end{array}\right. }, \end{aligned}$$

for $k^*\in (-(1-c_i-d_i)(1-P(X_i=1|\theta , \Gamma ^{1}_i)), \min _{i,j}{(d_i, c_ie^{\theta _j-b_i})}]$ leave the IRF of both the 1PL-GS and the 1PL models unchanged (Ogasawara, 2017, Theorem 5). Transformations (2) can also be further extended to the 1PL-GS model by considering an additional condition

5 $$\begin{aligned} (1-d_i^*)&= \frac{(1-d_i)e^{\theta }+\ell }{e^{\theta }+\ell } \end{aligned}$$

which actually transforms the slipping parameter $d_i^*$ into a function $d_i^*(\theta )$ of the ability (Ogasawara, 2017). Equation (5) might be of interest if the slipping parameter $d_i$ is assumed to be person-dependent, i.e., of the form $d_j$. Transformations (4) and (5) are, in the present context, not considered unidentifiability results but rather empirically indistinguishable alternative models since, in both cases, some left-side added parameters have become ability-dependent and are not constant anymore. Finally, it is worth mentioning that Ogasawara (2020) discussed additional identifiability restrictions for the 1PL-G model based on the minimization of the absolute value of the skewness of the $\theta ^*$ abilities. More in general, Ogasawara (2021) discussed the maximization of Fisher information, total score information, and total quasi-information to impose optimal restrictions that allow to resolve the identifiability of the 1PL-G model and of the general family of models given by Equation (3). As it will be shown in Sect. 4, the transformations (2) introduced by Maris and Bechger (2009), as well as the general case (3) and the special cases (4) and (5) introduced by Ogasawara (2017), or the other results on unidentifiability provided by Wu (2016), van der Linden and Barrett (2016), and San Martín (2016) can be derived from the general transformations describing the trade-off between the parameters $c_i$, $d_i$, and the entire $P(X_i=1|\theta ,\Gamma ^2_i)$ as a consequence of the forward- and backward-gradedness of the power set describing the local stochastic independence assumption in IRT between the different items. In order to show this, we first need to introduce KST.

## Identifiability in KST

### A Brief Introduction to KST

**Fig. 1:**
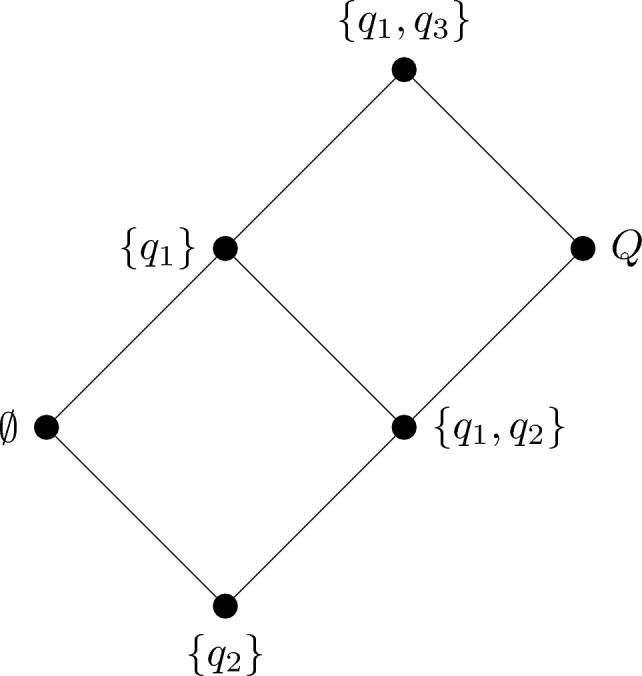
Example of a knowledge structure for a set of five items $Q=\{q_1, q_2,q_3\}$.

KST is a combinatorial and set-theoretical approach that classifies individuals by means of the collections of items that they can master in a given domain of knowledge (see, e.g., Falmagne and Doignon, 2011). Let *Q* be a nonempty set of items *q*, then a *knowledge state* $K\subseteq Q$ is a collection of problems $q\in Q$ that an individual is capable of mastering. A *knowledge structure* is a pair $(Q,\mathcal {K})$ where $\mathcal {K}$ is a family of subsets of *Q* that always includes the full domain *Q* and the empty set $\emptyset$. As an example, for the domain $Q=\{q_1,q_2,q_3\}$, a possible knowledge structure is defined by the collection

6 $$\begin{aligned} \mathcal {K}&=\{\emptyset , \{q_1\}, \{q_2\},\{q_1, q_2\},\{q_1, q_3\}, Q\}. \end{aligned}$$

and represents a situation in which item $q_1$ and $q_2$ can be mastered independently of each other, while item $q_3$ requires item $q_1$ to be mastered before it can be mastered. Knowledge structures are graphically displayed by Hasse diagrams as shown in Fig. 1, where each node is a different knowledge state.

Different properties of and relations between the knowledge states characterize different families of structures. For instance, if the structure is closed under union (i.e., $K,L\in \mathcal {K}$ implies $K\cup L \in \mathcal {K}$), the knowledge structure is called a *knowledge space*. Such a property is a natural requirement for substantive applications in learning as it implies that any item can be mastered at any time (provided, of course, that the prerequisite conditions for learning it are fulfilled). Another characteristic which is deemed a necessary requirement to learning is that items can be learned one at a time, that is, the structure is *well-graded* (for every state $K\in \mathcal {K}\setminus Q$ there is an item $q\in Q\setminus K$ such that $K\cup \{q\}\in \mathcal {K}$). If the structure is closed under union and well-graded, it is called a *learning space*. The knowledge structure shown in Fig. 1 can be easily verified to satisfy the requirements for a learning space (moreover, it is also closed under intersection).

Deterministic structures like (6) are made probabilistic by considering a probabilistic knowledge structure (PKS), i.e., a triple $(Q,\mathcal {K},\pi )$ in which $\pi :\mathcal {K}\rightarrow [0,1]$ is a probability distribution over the knowledge states $K\in \mathcal {K}$. Although usually in KST, the response pattern is denoted by $R\in 2^Q$, we will use here the notation $X\in 2^Q$ to uniform the notation with the IRT one. The probability of a given response pattern $X\in 2^Q$ in the data (i.e., solved and non-solved items) is given by:

7 $$\begin{aligned} P(X)&=\sum _{K\in \mathcal {K}}P(X|K)\pi (K), \end{aligned}$$

where one only needs to set the conditional probabilities *P*(*X*|*K*). The most widely used model is the Basic Local Independence Model (BLIM), in which the conditional probabilities *P*(*X*|*K*) are written as

8 $$\begin{aligned} P(X|K)&= \prod _{q\in X} \phi _{q,K} \prod _{q'\in Q\setminus X} (1-\phi _{q',K}), \end{aligned}$$

in which

9 $$\begin{aligned} \phi _{q,K}&= {\left\{ \begin{array}{ll} 1-\beta _q &{} \text {if}\,q\in K\\ \eta _q &{} \text {if}\,q\notin K. \end{array}\right. } \end{aligned}$$

where lucky guesses $\eta = \{\eta _{q}\}$ model correct observed responses even though an item is not in the knowledge state of an individual, whereas careless errors $\beta = \{\beta _{q}\}$ model incorrect observed responses even though an item is in the knowledge state of an individual.

### Unidentifiability of the BLIM

Given an arbitrary knowledge structure $\mathcal {K}$, some of the parameters in the set $\Gamma _\mathcal {K}=\{\pi ,\beta ,\eta \}$ used in the BLIM (8) can turn out to be unidentified as a consequence of a specific nature of the knowledge structure $\mathcal {K}$. For more detailed discussions, the reader is referred, for instance, to Spoto, Stefanutti, and Vidotto (2012; 2013), Heller (2017), Stefanutti, Spoto and Vidotto (2018), and Stefanutti and Spoto (2020). For the purpose of the present manuscript, it is sufficient to introduce the notions of forward- and backward-graded knowledge structure. In more details, a knowledge structure $(Q,\mathcal {K})$ is said to be *forward-graded* (FG) in an item *q* if $K\cup \{q\}\in \mathcal {K}$ for every $K\in \mathcal {K}$. Conversely, a knowledge structure $(Q,\mathcal {K})$ is said to be *backward-graded* (BG) in an item *q* if $K{\setminus } \{q\}\in \mathcal {K}$ for every $K\in \mathcal {K}$. In the FG (BG) case, joining (removing) an item *q* to (from) any state of the structure always yields another state of the structure. For instance, consider the structure given in example (6). It is FG in $q_1$, but not BG in that item (e.g., $\{q_1,q_3\}\setminus \{q_1\}=\{q_3\}$ is not a state). The structure is also both FG and BG in $q_2$. It is BG in $q_3$, but it is not FG in this item ($\emptyset \cup \{q_3\}=\{q_3\}$ is not a state). Finally, notice that the power set $\mathcal {K}=2^Q$ is both FG and BG in all items $q\in Q$ since any item can be both added to or removed from any state.

In order to describe the relation between FG and BG structures and identifiability, it is convenient to introduce the following collections of states:

10 $$\begin{aligned} \mathcal {K}_q&= \{K\in \mathcal {K}|\, q\in K\} \end{aligned}$$

11 $$\begin{aligned} \overline{\mathcal {K}}_q&= \{K\in \mathcal {K}|\, q\not \in K\} \end{aligned}$$

12 $$\begin{aligned} \mathcal {K}_{q}^-&= \{K\setminus \{q\}|\,K\in \mathcal {K}_q\} \end{aligned}$$

13 $$\begin{aligned} \overline{\mathcal {K}}_{q}^+&= \{K\cup \{q\}|\,K\in \overline{\mathcal {K}}_q\} \end{aligned}$$

Collection (10) is the collection of all states that contain a given item. Collection (11) is the complement in $\mathcal {K}$ of the former collection. Collection (12) is the collection resulting from removing the item *q* from all states in $\mathcal {K}_q$. Collection (13) is the collection resulting from adding the item *q* to all states in the complement of $\mathcal {K}_q$. Let *m* be the dimension of the parameter space of $\Gamma _\mathcal {K}=\{\eta ,\beta ,\pi \}$, in the KST literature (see, e.g., Stefanutti and Spoto, 2020) it has been established that, if the structure is FG in an item *q*, then for every $t\in \mathbb {R}$ there exists a transformation $f^t_q:\mathbb {R}^{m}\rightarrow \mathbb {R}^{m}$ that yields a new set of parameter values $\Gamma ^t_\mathcal {K}=\{\eta ',\beta ',\pi '\}$ given by $\Gamma ^t_\mathcal {K}=f^t_q(\Gamma _{\mathcal {K}})$, that is

14 $$\begin{aligned} {\left\{ \begin{array}{ll} \beta '_p = \beta _p\quad \text {for all } p\in Q \\ \eta '_p = \eta _p\quad \text {for all } p\in Q, p\ne q \\ \eta '_q = \eta _q +(1-\eta _q-\beta _q)(1-e^t)\\ \pi '(K) = {\left\{ \begin{array}{ll} \pi (K)+(1-e^{-t})\pi (K\setminus \{q\}) &{} \text {for all } K\in \overline{\mathcal {K}}^+_q \\ e^{-t}\pi (K) &{} \text {for all } K\in \overline{\mathcal {K}}_q \\ \pi (K) &{}\text {for all } K\in \mathcal {K}\setminus (\overline{\mathcal {K}}_q\cup \overline{\mathcal {K}}^+_q ) \end{array}\right. } \end{array}\right. } \end{aligned}$$

such that both sets of parameters $\Gamma _{\mathcal {K}}$ and $\Gamma ^t_{\mathcal {K}}$ result in the same *P*(*X*) as given by Equation (7), that is $P(X)=P(X|\Gamma ^t_{\mathcal {K}}) = P(X|f_q^t(\Gamma _{\mathcal {K}})) = P(X|\Gamma _{\mathcal {K}})$. If instead the structure is BG in an item *q*, then for every $t\in \mathbb {R}$ there exists a transformation $b^t_q:\mathbb {R}^{m}\rightarrow \mathbb {R}^{m}$ that yields a new set of parameter values $\Gamma ^t_\mathcal {K}=\{\eta ',\beta ',\pi '\}$ given by $\Gamma ^t_\mathcal {K}=b^t_q(\Gamma _{\mathcal {K}})$, that is

15 $$\begin{aligned} {\left\{ \begin{array}{ll} \eta '_p = \eta _p\quad \text {for all } p\in Q \\ \beta '_p = \beta _p\quad \text {for all } p\in Q, p\ne q \\ \beta '_q = \beta _q +(1-\eta _q-\beta _q)(1-e^t)\\ \pi '(K) = {\left\{ \begin{array}{ll} \pi (K)+(1-e^{-t})\pi (K\cup \{q\}) &{} \text {for all } K\in \mathcal {K}^-_q \\ e^{-t}\pi (K) &{} \text {for all } K\in \mathcal {K}_q \\ \pi (K) &{}\text {for all } K\in \mathcal {K}\setminus (\mathcal {K}_q\cup \mathcal {K}^-_q ) \end{array}\right. } \end{array}\right. } \end{aligned}$$

such that both sets of parameters $\Gamma _{\mathcal {K}}$ and $\Gamma ^t_{\mathcal {K}}$ result in the same *P*(*X*) as given by Equation (7), that is $P(X)=P(X|\Gamma ^t_{\mathcal {K}}) = P(X|b_q^t(\Gamma _{\mathcal {K}})) = P(X|\Gamma _{\mathcal {K}})$. As it will be shown in what follows, the set of transformations (14) and (15) allow to derive the IRT transformations (2) and as such they allow to describe the unidentifiability of 3PL and 4PL models (or more, in general, any IRF to which guessing and slipping parameters are left-side added) as a consequence of the forward- and backward- gradedness of the power set structure in all its items. In order to do so, one needs to first better detail the relation between the IRT and the KST frameworks. It is important to stress that forward- and backward-gradedness might, however, not be the only sources of unidentifiability in KST models. Trade-offs among $\eta$ and $\beta$ parameters can indeed also occur both in the presence and absence of forward- and backward-gradedness (see, e.g., Heller, 2017). A complete description of the unidentifiability problem in KST for an arbitrary structure has yet to be achieved. Nonetheless, as it will be discussed in the next subsection, the KST structure underlying IRT models is the power set, and such a structure is both FG and BG in all items. As it will be shown, the IRT results on unidentifiability can be fully traced back to these transformations even when a trade-off between the left-side added parameters occurs, without affecting the IRF, as in Equation (4). Finally, it is worthy to remark that the transformations (14) and (15) have been given for arbitrary values of a parameter $t\in \mathbb {R}$. In what follows, in order to distinguish FG and BG transformations, we will denote their parameters as $t_F$ and $t_B$, respectively. Although these parameters are defined over the entire $\mathbb {R}$, their actual domains are restricted by the domain of the parameters. When transformations (14) and (15) are restricted in such a way, they are called *inner transformations*, and their domains have been given by Stefanutti, Spoto and Vidotto (2018). For the backward case, it must hold $\beta '_q \in (0,1-\eta _q)$ and $\pi '(K)\in (0,1)$, which implies $t_B\in (\max {[\log {\frac{\pi (K\cup \{q\})}{\pi (K)+\pi (K\cup \{q\})}}]}, \log {\frac{1-\eta _q}{1-\beta _q-\eta _q}})$. For the forward case, it must hold $\eta '_q \in (0,1-\beta _q)$ and $\pi '(K)\in (0,1)$, which implies $t_F\in (\max {[\log {\frac{\pi (K{\setminus }\{q\})}{\pi (K)+\pi (K{\setminus }\{q\})}}]}, \log {\frac{1-\beta _q}{1-\beta _q-\eta _q}})$.

### The Simple Learning Model and the IRT-KST Relation

The present subsection summarizes results reported in Noventa et al. (2019). The interested reader is referred to the original source for a more comprehensive treatise. Large domains of items imply large numbers of parameters in the set $\Gamma _\mathcal {K}=\{\pi ,\beta ,\eta \}$. A possible approach for reducing the number of parameters involves constraining the distribution of knowledge-state probabilities $\pi$. An example of such an approach is provided by the Simple Learning Model (SLM, see, e.g., Falmagne and Doignon, 2011, p. 199) for learning spaces, that is

16 $$\begin{aligned} \pi (K) = \prod \limits _{q\in K} g_q \prod \limits _{q'\in K^{\mathcal {O}}} (1- g_{q'}) \end{aligned}$$

where $g_q\in (0,1)$ is the probability of mastering item *q* and where $K^{\mathcal {O}}$ is the *outer fringe* of the knowledge state *K*, which is given by

17 $$\begin{aligned} K^{\mathcal {O}}:=\{q\in Q\setminus K : K \cup \{q\}\in \mathcal {K}\}. \end{aligned}$$

Intuitively, the outer fringe is the set of items that can be learned next when moving from the knowledge state *K*. Hence, the SLM factorizes the probability of each state into the product of the probabilities of the items that have already been learned (i.e., those in *K*) and the complementary probabilities of the items that can be learned next (i.e., items in $K^{\mathcal {O}}$). Generalized versions of the SLM have been explored by Noventa, Heller and Stefanutti (2021). The relevance of the SLM (16) for the present manuscript is that it provides a generalized version of local stochastic independence in IRT so that, when the power set $\mathcal {K}=2^Q$ is considered, the combination of SLM and BLIM (8) yields exactly the likelihood of a 4-parameter IRT model in the presence of local independence. As formally shown by Noventa et al. (2019), both the state probability $\pi (K)$ and the item probability $g_q$ can be extended to encompass some latent variable $\theta \in \mathbb {R}$ (e.g., the ability of individuals), thus yielding, respectively, some functions $\pi (K|\theta )$ and $g_{q}(\theta )$. While the latter is the probability of a correct response to a given item, conditional to the value of a latent variable, and as such, it captures an IRF, the former provides a state probability conditional to the value of a latent variable, and it is called a state response function (SRF). It is worth mentioning that both IRFs and SRFs can be given both a fixed-effects or a random-effects interpretation. In the former case, the latent variable $\theta$ is replaced with an incidental parameter $\theta _j$ as discussed in Sect. 2, and the SRF $\pi (K|\theta _j)$ can be interpreted compatibly with a stochastic subject view, as the probability of an individual with a certain ability $\theta _j$ to be in the knowledge state $K\in \mathcal {K}$. If instead a random-effects interpretation is chosen, the SRF $\pi (K|\theta )$ can be interpreted compatibly with a random sampling view, as the proportion of individuals with ability $\theta$ that are in the knowledge state $K\in \mathcal {K}$. In the random-effects interpretation, the state probability $\pi (K)$ can then be obtained by marginalizing out the ability in the SRF, that is $\pi (K) = \int d\theta \pi (K|\theta )$.

Given then the previously defined IRFs and SRFs, one can reformulate the SLM (16) as a generalization of the IRT notion of (strong) local stochastic independence, that is,

18 $$\begin{aligned} \pi (K|\theta )&= \prod \limits _{q \in K} g_{q}(\theta )\prod \limits _{q' \in K^{\mathcal {O}}}(1- g_{q'}(\theta )). \end{aligned}$$

Intuitively, (18) generalizes local independence in that it allows for the presence of items that cannot be mastered from a given state because their prerequisites are not satisfied (i.e., they are not in the outer fringe). The traditional formulation of local independence is retrieved only if the knowledge structure is a power set, $\mathcal {K} = 2^Q$. Indeed, in such case, one can master or fail any of the items in $K^{\mathcal {O}}=Q\setminus K$ as required by local independence. Let $X_q=\chi _{K}(q)$ the indicator function that takes value one if $q\in K$ and zero if $q\notin K$, then one has

19 $$\begin{aligned} \pi (K|\theta )&= \prod \limits _{q\in K} g_q(\theta ) \prod \limits _{q'\in Q\setminus K} (1- g_{q'}(\theta ))= \prod \limits _{q\in Q} g_q(\theta )^{X_q}(1- g_{q}(\theta ))^{1-X_q} \end{aligned}$$

which for $g_q(\theta )=P(X_q=1|\theta )$ yields the traditional IRT definition of (strong) local stochastic independence. As a further consequence, the combination of SLM plus BLIM in the KST-IRT framework yields the likelihood of the 4-parameter IRT model. Indeed, let $\Gamma _\mathcal {K}=\{\eta , \beta , g\}$ be the KST set of parameters, then Equation (7) can be rewritten, by substitution of Equations (8) and (16), as

$$\begin{aligned} P(X|\theta , \Gamma _\mathcal {K})&= \sum _{K\in 2^Q}P(X|K)\pi (K|\theta )\\&= \sum _{K\in 2^Q}\prod _{q\in X} \phi _{q,K} \prod _{q'\in Q\setminus X} (1-\phi _{q',K}) \prod \limits _{q\in K} g_q(\theta ) \prod \limits _{q'\in Q\setminus K} (1- g_{q'}(\theta )) \end{aligned}$$

which can be shown (see, Noventa et al., 2019, Theorem 3 for a proof of the result) to be equivalent to

20 $$\begin{aligned} P(X|\theta , \Gamma _\mathcal {K})&= \prod \limits _{X_q} P(X_q=1|\theta , \Gamma _\mathcal {K})^{X_q}(1- P(X_q=1|\theta , \Gamma _\mathcal {K}))^{1-X_q} \end{aligned}$$

where

21 $$\begin{aligned} P(X_q=1|\theta , \Gamma _\mathcal {K})&= \eta _q+(1-\beta _q-\eta _q)g_q(\theta ) \end{aligned}$$

which is clearly equivalent to the likelihood of a 4-parameter IRT model, e.g., the 4PL in Equation (1), as soon as one identifies $\eta _q:= c_i$, $\beta _q:= d_i$, and $g_q(\theta ): =P(X_i=1|\theta ,\Gamma ^2_i)$ so that $\Gamma _\mathcal {K}:=\Gamma ^4_i$. It is important to remark that, as a consequence of the results summarized in the present section, traditional IRT models, which assume local stochastic independence, are, therefore, the power set case of a KST-IRT approach. Assuming any other structure except the power set amounts to not assume local stochastic independence, and it has been suggested to provide an alternative way of modeling local dependence between items (see, e.g., Noventa et al., 2019; Ye et al., 2023). Since the present work aims to recover traditional IRT unidentifiability results, the only knowledge structure considered is the power set, as it is the only structure equivalent to the traditional IRT models that assume local independence. Further considerations on the use of other structures are briefly given in the discussion section.

## Main Results

Since the knowledge structure naturally associated with the traditional IRT models is the power set $2^Q$, the study of unidentifiability for IRT models must be carried out within such a structure to provide comparable results to those in the literature. Section 4.1 derives the KST transformations associated with the SLM in the power set case, i.e., the transformations of the parameter $g_q$ which are implied by the FG and BG transformations of the parameter $\pi$. Section 4.2 applies the transformations derived in Sect. 4.1 (more precisely, their inverse) to an IRF $g_q(\theta )$ and shows that these capture a trade-off between the left-side added parameters and the 2-parameter IRFs. Finally, in Sect. 4.3, a logistic function is considered to recover the IRT results on unidentifiability summarized in Sect. 2. A couple of remarks are in order. Since KST uses *q* as a subscript to denote an arbitrary item, while IRT uses a subscript *i* to denote the item, and since clearly $q_i$ stands for the *i*-th item, we switch between notations *q*, $q_i$, and *i* based on need, as their meaning is understood from the context. In addition, in order to distinguish between an IRF like $P(X_i=1|\theta ,\Gamma ^4_i)$ in Equation (1), which contains left-side added parameters, and an IRF like $P(X_i=1|\theta ,\Gamma ^2_i)$, which does not contain left-side parameters and is nested within the former, we explicitly refer to the former as a 4-parameter IRF and to the latter as a 2-parameter IRF.

### Unidentifiability of the SLM Model in the Power Set Case

Since the power set is FG and BG in any item *q*, the following transformations of the parameters $\Gamma _{\mathcal {K}}=\{\eta ,\beta , g\}$ of the SLM can be obtained

22 $$\begin{aligned} {\left\{ \begin{array}{ll} \beta '_q = \beta _q+(1-\eta _q -\beta _q)(1-e^{t_B})&{}\\ \eta '_q = \eta _q+(1-\eta _q -\beta _q)(1-e^{t_F})&{} \\ g'_q = \frac{g_q+e^{t_F}-1}{e^{t_F}+e^{t_B}-1} &{} \\ g'_p = g_p, \quad \eta '_p = \eta _p, \quad \beta '_p = \beta _p &{}\text {for all } p\ne q, \end{array}\right. } \end{aligned}$$

where $t_B\in (\log {g_q}, \log {\frac{1-\eta _q}{1-\beta _q-\eta _q}})$ and $t_F\in (\log {(1-g_q)}, \log {\frac{1-\beta _q}{1-\beta _q-\eta _q}})$ with the additional condition that $e^{t_F}+e^{t_B}>1$. A proof of system (22) and the associated ranges for $t_F$ and $t_B$ are given in Appendix A. Since all items follow Equations (22) there are as many trade-off parameters $t_B$ and $t_F$ as there are items. Hence, it is convenient to denote them as $t_F^q$ and $t_B^q$ to highlight such a dependence. It is shown in the next subsection that the proliferation of trade-off parameters can be reduced in IRT if the mathematical forms of the 2- and 4-parameter IRFs are preserved. Before moving to the IRT case, further considerations on the KST case are provided. For the purposes of what follows, it is convenient to redefine the trade-off parameters as $r^q_T = 1-e^{t^q_T}$ for $T\in \{B,F\}$. In such a way, the transformations (22) can be rewritten as: 
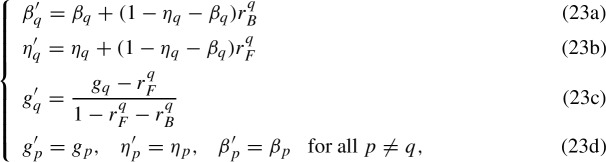
 where $r^q_B\in (-\frac{\beta _q}{1-\beta _q-\eta _q}, 1-g_q)$ and $r^q_F\in (-\frac{\eta _q}{1-\beta _q-\eta _q}, g_q)$ with the additional condition that $r^q_F+r^q_B < 1$. Equations (23a) and (23b) state that forward and backward gradedness respectively affect the lucky guess $\eta _q$ and the careless error $\beta _q$ by means of the trade-off parameters, $r^q_F$ and $r^q_B$. By considering the differences $\Delta \eta _q = \eta '_q-\eta _q$ and $\Delta \beta _q = \beta '_q-\beta _q$, these trade-off parameters can be interpreted as re-scaled differences of left-side added parameters, that is $r^q_F = \frac{\Delta \eta _q}{1-\eta _q-\beta _q}$ and $r^q_B = \frac{\Delta \beta _q}{1-\eta _q-\beta _q}$. Equation (23c) states that both forward- and backward-gradedness affect the item probability $g_q$ in the SLM by means of the same trade-off parameters. In particular, the difference $\Delta g_q = g'_q-g_q$ can be rewritten as:

24 $$\begin{aligned} \Delta g_q&= \frac{r^q_Bg_q-r^q_F(1-g_q) }{1-r^q_B-r^q_F}, \end{aligned}$$

which highlights how the change in the item probability parameter is an exchange between the probability of failing and that of succeeding, mediated by the trade-off parameters capturing the associated changes in the left-side added parameters. Hence, the KST transformations highlight a trade-off between the left-side added parameters and the *g*-parameters, which in the IRT case transfers into a trade-off between the left-side added parameters and the 2-parameter IRF. Equations (23d) state that all the parameters associated with any item $p\ne q$ are not affected by the forward- and backward-gradedness of the structure in *q*. In order to better understand the implications of transformations (22) for the IRT models, it is convenient to stress that they provide the solution to the following equation

25 $$\begin{aligned} \eta '_q +(1-\eta '_q-\beta '_q)g'_q = \eta _q +(1-\eta _q-\beta _q)g_q, \end{aligned}$$

which is clearly (minus the dependence on $\theta$) the unidentifiability condition of a 4-parameter model as in Sect. 2. By considering the differences $\Delta \eta _q$, $\Delta \beta _q$, and $\Delta g_q$, Equation (25) can be re-written as:

26 $$\begin{aligned} (1-g_q)\Delta \eta _q- g_q\Delta \beta _q + (1- \eta _q-\beta _q)\Delta g_q -(\Delta \eta _q+\Delta \beta _q)\Delta g_q = 0 \end{aligned}$$

and it is straightforward to verify that, by substitution of Equations (23a) and (23b), Equation (26) becomes Equation (24), which is indeed a consequence of Equation (23c). Although such a result is expected as transformations (23) are the solutions to Equation (25), this rewriting highlights that other solutions, which have been identified in the literature, are sub-cases of System (23). Although there are several potential sub-cases, up to our knowledge, only two have been considered in the IRT literature:

1. If $\Delta g_q =0$, then the *g*-parameter is constant under transformations (23), and one obtains
  27 $$\begin{aligned} (1-g_q)\Delta \eta _q - g_q \Delta \beta _q = 0 \quad \Leftrightarrow \quad \Delta \eta _q = \frac{g_q}{1-g_q}\Delta \beta _q \quad \Leftrightarrow \quad r^q_F = \frac{g_q}{1-g_q} r^q_B \end{aligned}$$
  that represents an exclusive trade-off between the conditional error parameters in KST, which was described by Heller (2017). There, the trade-off was obtained by assuming that $\pi (K)=\pi (K\cup \{q\})$, which corresponds to assume $1-g_q = g_q$ in the SLM. In the KST context, Equation (27) can be generalized to $\Delta \eta _q = \frac{\pi (K\cup \{q\})}{\pi (K)}\Delta \beta _q$ for all $K\in \overline{\mathcal {K}}_q$ and is therefore a sub-case of a structure which is both BG and FG in an item. This case is shown in Sect. 4.3 to correspond to the IRT case described by Ogasawara (2017, Theorem 5), and given in Sect. 2 by System (4).
2. Sub-cases of transformations (23) can be obtained by splitting Equation (26) into a system of more equations. For instance, one might consider the following split with associated solutions
  28 $$\begin{aligned} {\left\{ \begin{array}{ll} (1-g_q -\Delta g_q)\Delta \eta _q + (1- \eta _q)\Delta g_q = 0\\ (g_q+\Delta g_q)\Delta \beta + \beta _q\Delta g_q = 0 \end{array}\right. } \quad \Leftrightarrow {\left\{ \begin{array}{ll} \Delta \eta _q = - (1- \eta _q)\frac{\Delta g_q}{(1-g_q -\Delta g_q)}\\ \Delta \beta _q = -\beta _q\frac{\Delta g_q}{g_q +\Delta g_q} \end{array}\right. }, \end{aligned}$$
  which will be shown to correspond to the sub-case given in Sect. 2 by Equation (5).

### Application of the KST Transformations to the KST-IRT Case

According to the KST transformations (23), the 2-parameter IRF $g_q(\theta )$ is expected to trade-off with the left-side added parameters. However, such a trade-off can occur in two distinct situations: the first one, in which only the KST transformations (23) are applied; and the second one, in which additional assumptions are imposed such that the mathematical form of the 2-parameter IRFs and/or the 4-parameter IRFs is preserved. The former case yields empirically indistinguishable models, in which different values of the left-side added parameters are associated with different 2-parameter IRFs, but the parameters within the 2-parameter IRFs $g_q(\theta )$ are unaffected. The latter case yields instead a more nuanced situation in which the parameters $\theta$ and $\Gamma ^{2}_i$ within the 2-parameter IRFs $g_q(\theta )$ are affected by the transformations, and both empirical indistinguishability and unidentifiability can manifest themselves, depending on whether or not the transformations also preserve the mathematical form of the 4-parameter IRF.

#### Empirically Indistinguishable IRFs

Application of transformations (23) to a 4-parameter IRF yields the following system of equations 
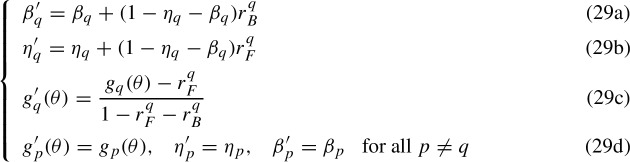
 in which a new set of 4-parameter IRFs is provided that yields the same probability of the responses. System (29) is a case of empirical indistinguishability as the mathematical forms of the 2-parameter IRFs $g_q(\theta )$ and $g'_{q}(\theta )$ are different. In order to discuss the IRT case, it is convenient to rewrite System (29) in a notation that is more consistent with the IRT notation used in Sect. 2 and to invert it to match the mathematical form of the results that can be found in the IRT literature on unidentifiability. Given then the previous system, it is convenient to invert Equations (29a) and (29b) to obtain the transformations

$$\begin{aligned} {\left\{ \begin{array}{ll} \eta _q = \frac{1}{1-r^q_B-r^q_F}[ (1-r^q_B)\eta '_q-(1-\beta '_q)r^q_F]\\ \beta _q = \frac{1}{1-r^q_B-r^q_F}[ (1-r^q_F)\beta '_q-(1-\eta '_q)r^q_B] \end{array}\right. } \end{aligned}$$

so that, once set $c_i:= \eta '_{q_i}$, $c^*_i:= \eta _{q_i}$, $d_i:= \beta '_{q_i}$, $d^*_i:= \beta _{q_i}$, $r^i_B:= r^{q_i}_B$, $r^i_F:= r^{q_i}_F$, and once identified the IRFs $P^*(X_i=1|\theta , \Gamma ^{2}_i):= g_{q_i}(\theta )$ and $P(X_i=1|\theta , \Gamma ^{2}_i):= g'_{q_i}(\theta )$ we can then set the general transformations for empirical indistinguishability in the IRT 4-parameter case as 
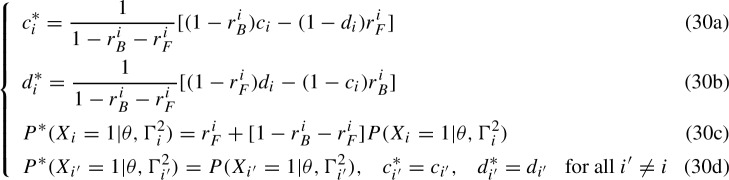
 and where $P^*(X_i=1|\theta , \Gamma ^{2}_i)$ and $P(X_i=1|\theta , \Gamma ^{2}_i)$ depend on the same set of parameters $\theta$ and $\Gamma ^{2}_i$ but have different mathematical form. Let $\Gamma ^{4*}_i=\{a_i,b_i,c^*_i,d^*_i\}$, the KST transformations yield the 4-parameter IRFs $P^*(X_i=1|\theta , \Gamma ^{4*}_i)$ that are empirically indistinguishable from the 4-parameter IRFs $P(X_i=1|\theta , \Gamma ^{4}_i)$, that is $P(X_i=1|\theta , \Gamma ^{4}_i) = P^*(X_i=1|\theta , \Gamma ^{4*}_i)$. Like in the KST case, there are as many trade-off parameters $r^i_F$ and $r^i_B$ as there are items. In passing, one might read the newly obtained $P^*(X_i=1|\theta , \Gamma ^{2}_i)$ in Equation (30c) themselves as 4-parameter IRFs in which the trade-off parameters act similarly to left-side added parameters but can attain negative values. System (30) holds in both fixed- and random-effects cases. In the fixed-effects case, the empirical indistinguishability concerns the 4-parameter IRFs $P^*(X_{ji}=1|\theta _j, \Gamma ^{4*}_i)$. In the random-effects case, the empirical indistinguishability can be interpreted in terms of either the likelihood of a response pattern *X* or of the marginal probability of a correct response to a given item. In the former case, it indeed holds that

$$\begin{aligned} P(X|\Gamma ^{4}_i)&= \int f(\theta ; \mu , \sigma ) \prod _{i = 1}^{|Q|}P(X_i|\theta , \Gamma ^{4}_i)d\theta \\&= \int f(\theta ; \mu , \sigma ) \prod _{i = 1}^{|Q|}P^*(X_i|\theta , \Gamma ^{4*}_i)d\theta = P^*(X|\Gamma ^{4*}_i) \end{aligned}$$

so that both sets of IRFs return the same likelihood of a pattern of responses. In the latter case, by local independence, the marginal probability $P(X_i=1|\Gamma ^{4}_i) = \sum _{X \in 2^Q, X_i =1} P(X|\Gamma ^{4}_i)$ of a correct response to the *i*-th item coincides with the marginalization of the 4-parameter IRFs, that is

$$\begin{aligned} P(X_i=1|\Gamma ^{4}_i)&= c_i+(1-c_i-d_i)\int f(\theta ; \mu ,\sigma )P(X_i|\theta , \Gamma ^{2}_i)d\theta \\&= c^*_i+(1-c^*_i-d^*_i)\int f(\theta ; \mu ,\sigma )P^*(X_i|\theta , \Gamma ^{2}_i)d\theta = P^*(X_i=1|\Gamma ^{4*}_i), \end{aligned}$$

and one can interpret Equation (30c) as a transformation of the marginal probability $P(X_i=1|\Gamma ^{2}_i)$, that is

$$\begin{aligned} P^*(X_i=1|\Gamma ^{2}_i)&= \int f(\theta ; \mu ,\sigma ) P^*(X_i=1|\theta , \Gamma ^{2}_i)d\theta = r^i_F+[1-r^i_B-r^i_F]P(X_i=1|\Gamma ^{2}_i). \end{aligned}$$

Finally, it is worth mentioning that in the random-effects case, the distribution of the latent trait $f(\theta ;\mu ,\sigma )$ is unaffected by the transformations (30), so the empirical indistinguishability only concerns the IRFs.

#### Preserving the Mathematical Form of the IRFs

The IRT results summarized in Sect. 2 can be obtained if System (30) is supplemented with the assumption that the mathematical forms of the IRFs must be preserved. Let us assume that only the mathematical form of the 2-parameter IRFs is preserved. The transformation (30c) must then imply changes in the parameters of the 2-parameter IRF. Namely, there exist $\theta ^*\in \mathbb {R}$ and $\Gamma ^{2*}_i=\{b^*_i, a^*_i\}$ such that one can replace $P^*(X_i=1|\theta , \Gamma ^{2}_i)$ with $P(X_i =1|\theta ^*, \Gamma ^{2*}_i)$ in Equation (30c), thus yielding the system 
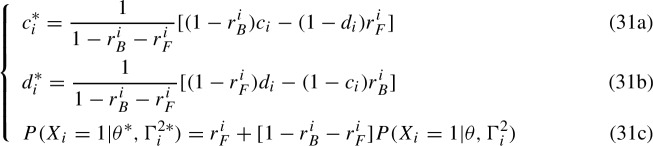
 such that $P(X_i=1|\theta , \Gamma ^{4}_i) = P(X_i=1|\theta ^*, \Gamma ^{4*}_i)$. Equation (30d) has been removed since changes propagate to all IRFs via the $\theta$ parameter. It follows that the trade-off between the left-side added parameters and the 2-parameter IRF, which was occurring in the system of transformations (30) for each item independently, is not anymore independent. That is, the trade-off parameters $r^i_F$ and $r^i_B$ must lose some if not all, their independence. In order to explore these implications, it is convenient to rewrite the 2-parameter IRF $g_{q_i}(\theta )$ as $g_{q_i}(\theta , \Gamma ^2_i)$ to underline the item parameters. Therefore, Equation (31c) can be rewritten as

32 $$\begin{aligned} g_{q_i}(\theta ^*,\Gamma ^{2*}_i)&= r^i_F+[1-r^i_B-r^i_F]g_{q_i}(\theta , \Gamma ^2_i), \end{aligned}$$

which establishes the general relation that the sets of parameters $(\theta ^*,\Gamma ^{2*}_i)$ and $(\theta , \Gamma ^{2}_i)$ must obey in order for the mathematical form of the 2-parameter IRFs to be preserved. Any transformation of the parameters that satisfy Equation (32) provides either empirically indistinguishable or unidentifiable transformations for the 4-parameter IRFs in System (31). However, not all possible transformations that satisfy (32) are also meaningful in IRT. For instance, one might want to restrict the set of all possible transformations to those in which the ability parameter $\theta ^*$ is independent of the difficulty parameter $b_i$ (same for $b^*_i$ and $\theta$). For this parameter independence to hold, one needs to transform the trade-off parameters $r^i_F$ and $r^i_B$ into trade-off functions $r^i_F(\theta , \Gamma ^2_i)$ and $r^i_B(\theta , \Gamma ^2_i)$ that cancel out the dependence on the undesired parameters. However, the functional forms of $r^i_F(\theta , \Gamma ^2_i)$ and $r^i_B(\theta , \Gamma ^2_i)$ must be set on a case-by-case basis as they depend on the specific choice of the 2-parameter IRF $g_{q_i}(\theta ,\Gamma ^{2}_i)$. As an example, let us consider the 1-parameter IRF $g_{q_i}(\theta , b_i) = \frac{G(\theta )}{G(\theta )+H(b_i)}$ of Equation (3), which was suggested by Ogasawara (2017) to generalize the 1PL-G model (for which $H=G=\exp$). By substitution of the IRF (3), Equation (32) becomes

33 $$\begin{aligned} \frac{G(\theta ^*)}{G(\theta ^*)+H(b^*_i)} = r^i_F+[1-r^i_B-r^i_F]\frac{G(\theta )}{G(\theta )+H(b_i)} = \frac{r^i_FH(b_i)+(1-r^i_B)G(\theta )}{G(\theta )+H(b_i)} \end{aligned}$$

that, after a little algebra, can be rewritten as

34 $$\begin{aligned} \frac{G(\theta ^*)}{H(b^*_i)} = \frac{r^i_FH(b_i)+(1-r^i_B)G(\theta )}{r^i_BG(\theta )+(1-r^i_F)H(b_i)} = \frac{G(\theta )+r^i_FH(b_i)-r^i_BG(\theta )}{H(b_i)-(r^i_FH(b_i)-r^i_BG(\theta ))}. \end{aligned}$$

Any solution to Equation (34) satisfies system (31). However, if one wants the transformations of $\theta$ and $b_i$ to be independent on the other parameter, as is the case in IRT, one needs to consider the solution

35 $$\begin{aligned} {\left\{ \begin{array}{ll} G(\theta ^*) = kG(\theta )+k(r^i_FH(b_i)-r^i_BG(\theta ))\\ H(b^*_i) = kH(b_i)-k(r^i_FH(b_i)-r^i_BG(\theta )) \end{array}\right. } \end{aligned}$$

for some $k\in \mathbb {R}$, and require that the term $\ell := r^i_FH(b_i)-r^i_BG(\theta )$ be a constant $\ell \in \mathbb {R}$. This requires the trade-off parameters $r^i_F$ and $r^i_B$ to become the trade-off functions

36 $$\begin{aligned} {\left\{ \begin{array}{ll} r^i_F(b_i) = \ell ^i_F H(b_i)^{-1}\\ r^i_B(\theta ) = -\ell ^i_B G(\theta )^{-1}\\ \ell = \ell ^i_F+\ell ^i_B \end{array}\right. } \end{aligned}$$

for some newly defined trade-off parameters $\ell ^i_F,\ell ^i_B\in \mathbb {R}$. The trade-off functions $r^i_F(b_i)$ and $r^i_B(\theta )$ remove the dependence of $\theta ^*$ and $b_i^*$ on, respectively, $b_i$ and $\theta$, thus yielding the system

37 $$\begin{aligned} {\left\{ \begin{array}{ll} \theta ^* = G^{-1}(kG(\theta )+k\ell )\\ b^*_i = H^{-1}(kH(b_i)-k\ell ) \end{array}\right. } \end{aligned}$$

that provides the transformations discussed by Ogasawara (2017; 2020) for the IRF (3). By combining together systems (31), (36), and (37), and by setting $k=1$ for practical purposes, one obtains for the 1-parameter model $P(X_i=1|\theta , b_i) = \frac{G(\theta )}{G(\theta )+H(b_i)}$ with guessing and slipping parameters, the system

38 $$\begin{aligned} {\left\{ \begin{array}{ll} c^*_i = \frac{1}{1-r^i_B(\theta )-r^i_F(b_i)}[ (1-r^i_B(\theta ))c_i-(1-d_i)r^i_F(b_i)]\\ d^*_i = \frac{1}{1-r^i_B(\theta )-r^i_F(b_i)}[ (1-r^i_F(b_i))d_i-(1-c_i)r^i_B(\theta )]\\ P(X_i=1|\theta ^*, b^*_i) = r^i_F(b_i)+[1-r^i_B(\theta )-r^i_F(b_i)]P(X_i=1|\theta , b_i) \\ G(\theta ^*) = G(\theta )+\ell , \quad H(b^*_i) = H(b_i)-\ell \\ r^i_F(b_i) = \ell ^i_F H(b_i)^{-1}, \quad r^i_B(\theta ) = -\ell ^i_B G(\theta )^{-1},\quad \ell = \ell ^i_F+\ell ^i_B \end{array}\right. } \end{aligned}$$

such that $P(X_i=1|\theta ,\Gamma ^{1,3}_i)=P(X_i=1|\theta ^*,\Gamma ^{1,3*}_i)$. Before moving to the logistic case, which yields the traditional IRT results, several general remarks are in order.

1. *The trade-off parameters* $\ell ^i_B$ *and* $\ell ^i_F$ *are item-dependent:* This is evident in the $\ell =0$ case, in which for each item a trade-off occurs exclusively between the left-side added parameters without involving the 2-parameter IRF. Indeed, for $\ell =0$ it holds $\theta ^* = \theta$ and $b^*_i = b_i$ so that $g_{q_i}(\theta ^*, b^*_i)=g_{q_i}(\theta , b_i)$, but one can still have $\ell ^i_B+\ell ^i_F=0$, that is $r^i_F(b_i)H(b_i)=r^i_B(\theta )G(\theta )$. This corresponds to the KST case in which $\Delta g_q = 0$ and $\Delta \eta _q = \frac{g_q}{1-g_q}\Delta \beta _q$ considered in Equation (27) of Sect. 4.1 and in the present case yields the trade-off $\Delta c_i = \frac{G(\theta )}{H(b_i)}\Delta d_i$. Further details are given in Sect. 4.3 for the logistic case.
2. *Alternative sets of transformations*: The mathematical form of the trade-off functions does not only depend on the mathematical form of the IRF but also on how the IRF is written. For instance, in System (38), the dependence on $\theta$ and $b_i$ of the trade-off functions can be reversed by rewriting the 1-parameter IRF as $g_{q_i}(\theta , b_i) = \frac{H(b_i)^{-1}}{G(\theta )^{-1}+H(b_i)^{-1}}$ and adapting the derivation accordingly to obtain
  39 $$\begin{aligned} {\left\{ \begin{array}{ll} c^*_i = \frac{1}{1-r^i_B(b_i)-r^i_F(\theta )}[ (1-r^i_B(b_i))c_i-(1-d_i)r^i_F(\theta )]\\ d^*_i = \frac{1}{1-r^i_B(b_i)-r^i_F(\theta )}[ (1-r^i_F(\theta ))d_i-(1-c_i)r^i_B(b_i)]\\ P(X_i=1|\theta ^*, b^*_i) = r^i_F(\theta )+[1-r^i_B(b_i)-r^i_F(\theta )]P(X_i=1|\theta , b_i) \\ G(\theta ^*)^{-1} = G(\theta )^{-1}-\ell , \quad H(b^*_i)^{-1} = H(b_i)^{-1}+\ell \\ r^i_F(\theta ) = \ell ^i_F G(\theta ), \quad r^i_B(b_i) = -\ell ^i_B H(b_i),\quad \ell = \ell ^i_F+\ell ^i_B \end{array}\right. } \end{aligned}$$
  that provides an alternative version of transformations (38) based on the same trade-off parameters $\ell ^i_F$ and $\ell ^i_B$ but with different trade-off functions and transformations. Identifiability conditions need to constrain these alternative sets of transformations. Although in line of principle, there might be many ways of rewriting an IRF, in practice, there are but a few of them if parameter independence is required as in System (35). For instance, the form $g_{q_i}(\theta , b_i) =\frac{G(\theta )H(b_i)^{-1}}{1+G(\theta )H(b_i)^{-1}}$ would defy such an independence. Most of all, there are no alternative versions of the IRF that allow the trade-off functions to be both ability-independent; hence, the transformed left-side added parameters are not constants (see Remark 3 below). The only exception occurs if all individuals have the same value of ability (see Remark 4).
3. *Empirical indistinguishability vs. unidentifiability:* As long as any of the trade-off functions is ability-dependent, the transformations (38) and (39) yield empirically indistinguishable 4-parameter IRFs with ability-dependent left-side added parameters $c^*_i(\theta , b_i)$ and $d^*_i(\theta , b_i)$. The only exception occurs if all individuals have the same value of ability so that $c^*_i$ and $d^*_i$ can be treated as constants. Let us assume that this is not the case (see Remark 4) and that there are at least two individuals with different abilities (the random-effects case is implicitly considered to have infinite values for the ability). If one assumes that not only the mathematical form of the 2-parameter IRF but also the mathematical form of the 4-parameter IRF must be preserved, then $c^*_i$ and $d^*_i$ must be constant values and the trade-off parameters $\ell ^i_T$ with $T\in \{B,F\}$ associated with the ability-dependent trade-off functions $r^i_T(\theta )$ must be equal to zero. In the case of System (38), this yields a restriction $\ell ^i_B=0$, which implies $d^*_i=d_i$ and $c^*_i = \frac{c_i-(1-d_i)r^i_F(b_i)}{1-r^i_F(b_i)}$, for all items. In other words, according to transformations (38), either $d^*_i=d_i$ or the left-side added parameters depend on the ability. The empirical indistinguishability is thus removed, and one is left with the unidentifiability of the guessing parameters. In addition, if $\ell ^i_B=0$ then $\ell ^i_F = \ell$ for all items. The trade-off functions $r^i_F(b_i) = \ell H^{-1}(b_i)$ still differ from item to item but have a common parameter $\ell$ that needs to be constrained to solve unidentifiability (e.g., set $c_1=c^*_1=0$ in a reference item). A similar reasoning holds for System (39). It thus appears that the identification issue of the 4-parameter models is one of empirical indistinguishability. Once the left-side added parameters are required to be constant in the presence of at least two individuals with different abilities, one is left with the unidentifiability issue of one of these parameters. Section 4.3 shows how these results relate to those already discussed in the literature for the logistic family of models.
4. *The equal abilities case:* This sub-case was first highlighted by van der Linden and Barrett (2016) in the context of the 3PL, but is of general relevance. When all individuals in a fixed-effects 4-parameter model have exactly the same value of ability, any set of transformations like (38) or (39) provides a constant reparameterization of the left-side added parameters. Similarly, the exclusive trade-off discussed in Remark 1 between the left-side added parameters becomes a constant trade-off. In the equal abilities case, the identification problem can thus be considered one of unidentifiability and not one of empirical indistinguishability. Hence, to enforce a restriction like $\ell ^i_T=0$ for $T\in \{B,F\}$, it is not sufficient to require the left-side added parameters to always be constant values, as discussed in Remark 3 above, and one needs at least two individuals with different abilities to be available.
5. *There are two independent sources of non-identification:* The first source of non-identification is the well-known unidentifiability of the 2-parameter IRF $g_{q_i}(\theta , \Gamma ^2_i)$. The second source is the trade-off between the left-side added parameters and the 2-parameter IRF $g_{q_i}(\theta , \Gamma ^2_i)$, which occurs independently of the mathematical form of the latter and is captured by System (31). As an example, let us consider the generic 1-parameter IRF $g_{q_i}(\theta , b_i) = \frac{G(\theta )}{G(\theta )+H(b_i)}$ and let us rewrite Equation (33) as
  40 $$\begin{aligned} g_{q_i}(\theta ^*, b^*_i) = g_{q_i}(\theta , b_i)+ \frac{r^i_F(b_i)H(b_i)-r^i_B(\theta )G(\theta )}{H(b_i)+G(\theta )} = g_{q_i}(\theta , b_i)+\frac{\ell }{H(b_i)+G(\theta )} \end{aligned}$$
  where the term $\frac{\ell }{H(b_i)+G(\theta )}$ captures the change $\Delta g_{q}$ as it was discussed in Equation (24) for the KST case in Sect. 4.1. The first source of non-identification is that one can multiply both numerator and denominator of the generic 1-parameter IRF $g_{q_i}(\theta , b_i) = \frac{G(\theta )}{G(\theta )+H(b_i)}$ by the same constant *k* without affecting the IRF (if $H=G=\exp$ this is the unidentifiability of the 1PL model). The second source of non-identification is the trade-off between the 1-parameter IRF and the left-side added parameters, which captures the change in the whole IRF and depends on $\ell$, $H(b_i)$, and $G(\theta )$. The second source is independent of the first one since multiplying both numerator and denominator by the same constant *k* on both sides of Equation (40) leaves all terms unaffected as $\ell$ scales to $k\ell$ as in System (37).
6. *Fixed- and random-effects:* The previous results hold for both fixed- and random-effects specifications. In the fixed-effects case, the incidental parameter $\theta _j$ replaces $\theta$, and System (31) provides a general result for the non-identification of the 4-parameter IRFs. In the random-effects case, the statistical model consists of both the IRF and the distribution of the ability, and as such, the identification problem is always one of empirical indistinguishability. Indeed, although both the mathematical forms of the 2- and 4-parameter IRFs can be constrained, thus removing, as discussed above, the problem of empirical indistinguishability and leaving only a problem of unidentifiability for the 4-parameter IRFs, the distribution $f^*(\theta ^*)$ of the transformed values $\theta ^*$ in general does not coincide with $f(\theta ; \mu , \sigma )$. Rather, as long as the IRF $g_{q_i}$ is differentiable, the change of variables formula yields the distribution
  41 $$\begin{aligned} f^*(\theta ^*)&= f\left( g_{q_i}^{-1}\left( \frac{g_{q_i}(\theta ^*)-r^i_F}{1-r^i_F-r^i_B}\right) ;\sigma , \mu \right) \frac{d}{d\theta ^*}g_{q_i}^{-1}\left( \frac{g_{q_i}(\theta ^*)-r^i_F}{1-r^i_F-r^i_B} \right) , \end{aligned}$$
  such that the different statistical models are observationally equivalent, that is
  $$\begin{aligned} P(X|\Gamma ^{4}_i)&= \int f(\theta ; \mu , \sigma ) \prod _{i = 1}^{|Q|}P(X_i|\theta , \Gamma ^{4}_i)d\theta \\&= \int f^*(\theta ^*) \prod _{i = 1}^{|Q|}P(X_i|\theta ^*, \gamma ^{4*}_i)d\theta ^* = P(X|\Gamma ^{4*}_i). \end{aligned}$$
  Hence, in the random-effects case, even if the identification problem of the 4-parameter IRFs is reduced to one of unidentifiability, the statistical models are empirically indistinguishable. However, resolving the unidentifiability of the 4-parameter IRFs also resolves the empirical indistinguishability.
7. *Sufficient and necessary conditions:* For all items, the condition $\ell ^i_B=\ell ^i_F=0$ occurs if and only if it holds that $c^*_i=c_i$ and $d^*_i=d_i$. Hence the condition is both necessary and sufficient to eliminate the trade-off between the left-side added parameters and the 2-parameter IRF for both fixed- and random-effects specifications. Alternative versions of System (31), based on alternative versions of the same IRF, are solved by the same condition. Therefore, any identifiability constraint that sets $\ell ^i_B=\ell ^i_F=0$ for all alternative systems erases this source of non-identification. However, full resolution of the unidentifiability of an IRT model requires to also provide a) the identifiability conditions for the 2-parameter IRF and b) the conditions on the minimal numbers of individuals and/or items that are required to provide enough equations to identify the parameter values. These latter depend on the identified parametrization associated with the observed outcomes. Let *N* be the number of individuals, |*Q*| the number of items, *I* the number of constraints imposed to reduce unidentifiability, and *n* the number of parameters per item. In the fixed-effects case, the identified parametrization consists of mutually independent Bernoulli distributions capturing the *N*|*Q*| response probabilities; hence, one needs to satisfy the condition $N|Q|\ge n|Q|+N-I$. In the random-effects case, the identified parametrization consists of a Multinomial distribution capturing the $2^{|Q|}-1$ independent patterns of responses, hence one needs to satisfy the condition $2^{|Q|}-1\ge n|Q|+2-I$, where the additional term 2 comes from the location and scale parameters of the distribution $f(\theta ; \mu , \sigma )$ of the abilities.

### The IRT logistic case

The IRT logistic case assumes the IRF $g_{q_i}(\theta , \Gamma ^2_i)=\frac{e^{a_i(\theta -b_i)}}{1+e^{a_i(\theta -b_i)}}$ so that System (30) becomes 
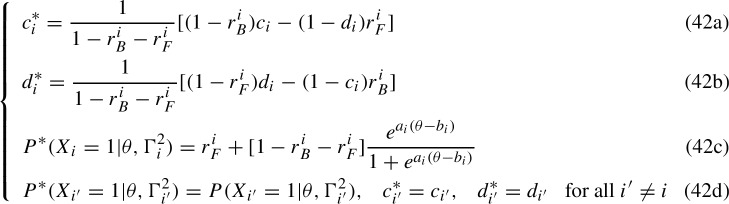
 such that $P(X_i=1|\theta ,\gamma _i^{4})=P^*(X_i=1|\theta ,\gamma _i^{4*})$ and in which the trade-offs due to the BG and FG in an item neither affects the IRFs of the other items nor the parameters within the 2-parameter IRFs. These are empirically indistinguishable IRFs. If one assumes that the mathematical form of the 2-parameter IRFs must also be preserved, then system (42) takes the form 
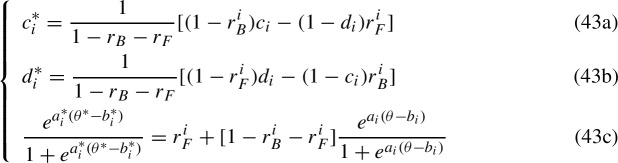
 such that $P(X_i=1|\theta , \Gamma ^{4}_i) = P(X_i=1|\theta ^*, \Gamma ^{4*}_i)$. Equation (43c) replaces Equation (42c), and is indeed a special case of Equation (32) establishing the relation between the different sets of parameters $(\theta , \Gamma ^2_i)$ and $(\theta ^*, \Gamma ^{2*}_i)$. Following the same rationale applied in Sect. 4.2, Equation (42d) has been removed since changes from $\theta$ to $\theta ^*$ affect all IRFs simultaneously. Equation (43c) can be rewritten as

44 $$\begin{aligned} \frac{e^{a^*_i\theta ^*}}{e^{a^*_ib^*_i}} = \frac{r^i_F e^{a_ib_i}+(1-r^i_B)e^{a_i\theta }}{r^i_Be^{a_i\theta }+(1-r^i_F)e^{a_ib_i}} = \frac{e^{a_i\theta }+(r^i_F e^{a_ib_i}-r^i_Be^{a_i\theta })}{e^{a_ib_i}-(r^i_Fe^{a_ib_i}-r^i_Be^{a_i\theta })}, \end{aligned}$$

which is indeed Equation (34) in which one sets $H=G=\exp$, and the discrimination parameters are not set to one. The IRT transformations are the inverse of the KST ones, with the difference that the difficulty, discrimination, and ability parameters are absorbed directly within the trade-off parameters $r^i_T$ with $T\in \{B,F\}$ that become general trade-off functions $r^i_T(\theta , \Gamma ^2_i)$. Traditional IRT results for unidentifiability, like the transformations associated with a logistic IRF and discussed by Maris and Bechger (2009), are then sub-cases of the solution of Equation (44) given by setting $\ell = r^i_Fe^{a_ib_i}-r^i_Be^{a_i\theta }$, which yields a more general set of transformations that captures the empirical indistinguishability of the 4PL, that is

45 $$\begin{aligned} {\left\{ \begin{array}{ll} c_i^* = \frac{1}{1-r^i_B(\theta , a_i)-r^i_F(\Gamma _i^2)}[(1-r^i_B(\theta , a_i))c_i-r^i_F(\Gamma _i^2)(1-d_i)]\\ d^*_i = \frac{1}{1-r^i_B(\theta , a_i)-r^i_F(\Gamma _i^2)}[(1-r^i_F(\Gamma _i^2))d_i-r^i_B(\theta , a_i)(1-c_i)]\\ P(X_i=1|\theta ^*, \Gamma ^{2*}_i) = r^i_F(\Gamma _i^2)+[1-r^i_B(\theta , a_i)-r^i_F(\Gamma _i^2)]P(X_i=1|\theta , \Gamma ^2_i) \\ e^{a_i^*\theta ^*}= e^{a_i\theta }+\ell , \quad e^{a_i^*b_i^*}= e^{a_ib_i}-\ell \\ r^i_B(\theta , a_i)=-\ell ^i_Be^{-a_i\theta }, \quad r^i_F(\Gamma _i^2)= \ell ^i_Fe^{-a_ib_i}, \quad \ell =\ell ^i_B+\ell ^i_F \end{array}\right. } \end{aligned}$$

where $\ell ^i_B, \ell ^i_F\in \mathbb {R}$ are such that $\ell \in [-e^{\min _{i,j}{(a_i\theta _j)}},e^{\min _i{(a_ib_i)}})$ for a fixed-effects model or $\ell \in [0,e^{\min _i{(a_ib_i)}})$ for a random-effects model. System (45) allows to recover and generalize the unidentifiability results discussed in Sect. 2. Results hold for both fixed- and random-effects specifications as they provide both sufficient and necessary conditions to remove the trade-off between the left-side added parameters and the 2PL. The identifiability conditions of the 2PL are well known (e.g., $b_1=0$ and $a_1=1$). Further conditions on the minimal number of individuals and items are also addressed. In the random-effects case, the problem of empirical indistinguishability involves also the transformed distribution (41) of the ability, which in the logistic case corresponds to a 3-parameter log-normal distribution if $f(\theta ; \sigma , \mu )$ is a normal distribution.

#### Unidentifiability of the 1PL-G and of the 1PL-S Models

The unidentifiability transformations (2) given by Maris and Bechger (2009) for the 1PL-G model $P(X_i=1|\theta , \Gamma ^{1,3}_i)$ are obtained by setting in System (45) that $d_i=0$, that all discrimination parameters are equal $a_i=a$ ($a=1$ without loss of generality), and that $\ell ^i_B=0$ for all items. This yields the system

46 $$\begin{aligned} {\left\{ \begin{array}{ll} c^*_i = \frac{c_i - r^i_F(b_i)}{1-r^i_F(b_i)}\\ P(X_i=1|\theta ^*,b^*_i) = r^i_F(b_i)+(1-r^i_F(b_i))P(X_i=1|\theta , b_i)\\ e^{\theta ^*}= e^{\theta }+\ell , \quad e^{b_i^*}= e^{b_i}-\ell \\ r^i_F(b_i)= \ell e^{-b_i} \end{array}\right. } \end{aligned}$$

such that $P(X_i=1|\theta , \Gamma ^{1,3}_i)=P(X_i=1|\theta ^*, \Gamma ^{1,3*}_i)$. By means of the trade-off functions $r^i_F(b_i)$, different items can have different trade-offs between the guessing parameter and the 1-parameter IRF. However, since the trade-off parameter $\ell ^i_F=\ell$ is common to all items, the identifiability of the 1PL-G model is ensured as soon as any condition that sets $\ell =0$ is imposed. A traditional condition that holds for both fixed- and random-effects models (see, e.g., San Martín, 2016) is to fix the guessing parameter of a reference item to a given value, which is typically zero (e.g., $c_1 = c^*_1 = 0$). Additional identifiability conditions have been discussed and summarized, for instance, in San Martín (2016), and are those associated to the unidentifiability of the 1PL model (e.g., fixing the difficulty of the reference item to $b_1=0$), and to the required minimal numbers of persons and/or items, which is $N\ge 2$ and $|Q|\ge 2$ for the fixed-effects 1PL-G model and $|Q|\ge 3$ for the random-effects 1PL-G model. Notice that rewriting the 1PL model in the alternative form $g_{q_i}(\theta , b_i)=\frac{e^{-b_i}}{e^{-b_i}+e^{-\theta }}$ yields an alternative set of transformations

47 $$\begin{aligned} {\left\{ \begin{array}{ll} c^*_i = \frac{c_i - r^i_F(\theta )}{1-r^i_F(\theta )}\\ P(X_i=1|\theta ^*, b^*_i) = r^i_F(\theta )+(1-r^i_F(\theta ))P(X_i=1|\theta , b_i)\\ e^{-\theta ^*}= e^{-\theta }-\ell , \quad e^{-b_i^*}= e^{-b_i}+\ell \\ r^i_F(\theta )= \ell e^{\theta } \end{array}\right. } \end{aligned}$$

such that $P(X_i=1|\theta , \Gamma ^{1,3}_i)=P(X_i=1|\theta ^*, \Gamma ^{1,3*}_i)$ and it is empirically indistinguishable from the 1PL-G model but has ability-dependent guessing parameters. This identification issue is resolved by assuming that the mathematical form of the 1PL-G model is preserved. If $c^*_i$ is assumed to be a constant, and there are at least two individuals with different abilities, then it must hold $\ell =0$ in System (47), which implies $c^*_i=c_i$. Besides, having at least two individuals with different abilities also solves the equal abilities sub-case. Finally, the same reasoning can be followed for the 1PL-S model $P(X_i=1|\theta , \Gamma ^{1,4}_i)$, which is obtained by setting in System (45) that $c_i=0$, that all discrimination parameters are equal $a_i=a$ ($a=1$ without loss of generality), and that $\ell ^i_F=0$ for all items, thus yielding the system of transformations

48 $$\begin{aligned} {\left\{ \begin{array}{ll} d^*_i = \frac{d_i-r^i_B(\theta )}{1-r^i_B(\theta )}\\ P(X_i=1|\theta ^*, b^*_i) = [1-r^i_B(\theta )]P(X_i=1|\theta , b_i) \\ e^{\theta ^*}= e^{\theta }+\ell , \quad e^{b_i^*}= e^{b_i}-\ell \\ r^i_B(\theta )=-\ell e^{-\theta } \end{array}\right. } \end{aligned}$$

such that $P(X_i=1|\theta , \Gamma ^{1,4}_i)=P(X_i=1|\theta ^*, \Gamma ^{1,4*}_i)$. As in the System (47), an identifiability condition is given by assuming that the mathematical form of the 1PL-S model is preserved while having two individuals with different abilities. By rewriting the 1PL model in the alternative form $g_{q_i}(\theta , b_i)=\frac{e^{-b_i}}{e^{-b_i}+e^{-\theta }}$, one obtains the alternative version

49 $$\begin{aligned} {\left\{ \begin{array}{ll} d^*_i = \frac{d_i-r^i_B(b_i)}{1-r^i_B(b_i)}\\ P(X_i=1|\theta ^*, b^*_i) = [1-r^i_B(b_i)]P(X_i=1|\theta , b_i) \\ e^{-\theta ^*}= e^{-\theta }-\ell , \quad e^{-b_i^*}= e^{-b_i}+\ell \\ r^i_B(b_i)=-\ell e^{b_i} \end{array}\right. } \end{aligned}$$

in which one needs to assume, for instance, $d_1=d^*_1=0$ to eliminate the trade-off between the slipping parameter and the 1-parameter IRF. These results are consistent with the extant literature.

#### Unidentifiability of the 3PL Model

The 3PL model differs from the 1PL-G model only in the fact that the discrimination parameters are not constrained to be equal. The transformations for the 3PL model are thus obtained from System (45) by assuming that $d_i=0$, and that $\ell ^{i}_B=0$ for all items, so that one obtains the system

50 $$\begin{aligned} {\left\{ \begin{array}{ll} c_i^* = \frac{c_i-r^i_F(\Gamma _i^2)}{1-r^i_F(\Gamma _i^2)}\\ P(X_i=1|\theta ^*, \Gamma _i^{2*}) = r^i_F(\Gamma _i^2)+[1-r^i_F(\Gamma _i^2)]P(X_i=1|\theta , \Gamma _i^2) \\ e^{a_i^*\theta ^*}= e^{a_i\theta }+\ell , \quad e^{a_i^*b_i^*}= e^{a_ib_i}-\ell \\ r^i_F(\Gamma _i^2)= \ell e^{-a_ib_i} \end{array}\right. } \end{aligned}$$

such that $P(X_i=1|\theta , \Gamma ^{3}_i)=P(X_i=1|\theta ^*, \Gamma ^{3*}_i)$. By setting $\ell ^{i}_B=0$, one has $\ell ^{i}_F=\ell$ for all items, and therefore, only one parameter captures the trade-off between the guessing parameter and the 2PL model. However, contrary to the 1PL-G model, the dependence on $a_i$ in the transformation of $\theta$ in System (50) implies that, if there are at least two items with different discrimination parameters, that is $a_i\ne a_{i'}$, then the trade-off parameter $\ell$ must be equal to zero. Indeed, only if all discrimination parameters are equal to each other can they be absorbed within a unit re-scaling of $\theta ^*$, which belongs to the unidentifiability of the 2PL itself, otherwise, they would imply that the transformation of $\theta$ is item-dependent, which is not acceptable. In the alternative, one can notice that the ratio of the transformed discrimination parameters

$$\begin{aligned} \frac{a_i^*}{a^*_{i'}}&= \frac{\log {(e^{a_i\theta }+\ell )}}{\log {(e^{a_{i'}\theta }+\ell )}} \end{aligned}$$

is constant for all values of $\theta$ if and only if $\ell = 0$. This coincides with the results obtained by Wu (2016) that consider a necessary condition for the fixed-effects 3PL to have at least two items with different discrimination parameters. This condition eliminates the trade-off between the guessing parameter and the 2PL in both fixed- and random-effects models. Notice, however, that if one assumes that all individuals have the same value of the ability, then the ratio $\frac{a_i^*}{a^*_{i'}}$ is always constant independently on $\ell$ so unidentifiability is restored, which is exactly the sub-case discussed by van der Linden and Barrett (2016). Hence, at least two individuals with different values of ability are also needed in the fixed-effects case. This is also consistent with the results obtained by Wu (2016), which consider a necessary condition for the 3-PL to have at least four individuals with different abilities. Two of these individuals are needed to solve the unidentifiability due to the trade-off between the guessing parameter and the 2PL, while the other two are due to the minimum number of individuals required to have identifiability. Given indeed $|Q|=2$ and considering $I=2$ identification constraints for the unidentifiability of the 2PL (e.g., $b_1=0$ and $a_1=1$ for the reference item), the condition $N|Q|\ge n|Q|+N-I$ yields $2N\ge 6+N-2$ that is $N\ge 4$ as given by Wu (2016). Finally, the identifiability constraints can also be given for the random-effects 3PL, which appears to require a) the identification constraints for the unidentifiability of the 2PL, and b) at least two items with different discrimination parameters out of a total of $|Q|\ge 4$ items available. The last value follows from the condition $2^{|Q|}-1\ge 3|Q|+2-I$ that for $I=2$ yields $2^{|Q|}\ge 3|Q|+1$ that is $|Q|\ge 4$. It thus appears that, contrary to the 1PL-G model, the fixed- and random-effects 3PL models do not require to set any guessing parameter to a reference value to be identified as long as there are at least two items with different discrimination parameters and, in the fixed-effects case, at least two individuals with different abilities.

#### Unidentifiability of the 4PL Model

The transformations associated with the 4PL are given by System (45). If one rewrites the 2PL as $g_{q_i}(\theta , \Gamma ^2_i)=\frac{e^{-a_ib_i}}{e^{-a_ib_i}+e^{-a_i\theta }}$, an alternative set of transformations can be obtained, which is given by:

51 $$\begin{aligned} {\left\{ \begin{array}{ll} c_i^* = \frac{1}{1-r^i_B(\Gamma _i^2)-r^i_F(\theta , a_i)}[(1-r^i_B(\Gamma _i^2))c_i-r^i_F(\theta , a_i)(1-d_i)]\\ d^*_i = \frac{1}{1-r^i_B(\Gamma _i^2)-r^i_F(\theta , a_i)}[(1-r^i_F(\theta , a_i))d_i-r^i_B(\Gamma _i^2)(1-c_i)]\\ P(X_i=1|\theta ^*, \Gamma ^{2*}_i) = r^i_F(\theta , a_i)+[1-r^i_B(\Gamma _i^2)-r^i_F(\theta , a_i)]P(X_i=1|\theta , \Gamma ^2_i) \\ e^{-a_i^*\theta ^*}= e^{-a_i\theta }-\ell , \quad e^{-a_i^*b_i^*}= e^{-a_ib_i}+\ell \\ r^i_B(\Gamma _i^2)=-\ell ^i_B e^{a_ib_i}, \quad r^i_F(\theta , a_i)= \ell ^i_Fe^{a_i\theta }, \quad \ell =\ell ^i_B+\ell ^i_F \end{array}\right. } \end{aligned}$$

and that reverses the dependence on the ability parameter in the trade-off functions. It can be shown that for both Systems (45) and (51), almost the same constraints of the 3PL model are needed. Indeed, like in the 3PL model case, given at least two items with different discrimination parameters and two individuals with different abilities, then it follows that $\ell =0$. As it was previously discussed, the fact that $\ell =0$ does not generally resolve empirical indistinguishability in the 4PL model. Let us consider System (45) without loss of generality. Given $\ell =0$ it might still hold $\ell ^i_F=-\ell ^i_B$, that is $r^i_Fe^{a_ib_i}=r^i_be^{a_i\theta }$. This corresponds to the KST case in which $\Delta g_q = 0$ and $\Delta \eta _q = \frac{g_q}{1-g_q}\Delta \beta _q$ considered in Equation (27) of Sect. 4.1. Specifically, in the IRT case, one has the trade-off

52 $$\begin{aligned} c^*_i - c_i = (d^*_i-d_i)\frac{P(X_i=1|\theta , \Gamma ^{2}_i)}{1-P^*(X_i=1|\theta , \Gamma ^{2}_i)} = (d^*_i-d_i)e^{a_i(\theta -b_i)}, \end{aligned}$$

and if one sets $d^*_i = d_i-k^*$ so that $(1-d^*_i) = (1-d_i)+k^*$ for some $k^*\in \mathbb {R}$, it follows $c^*_i = c_i -ke^{a_i(\theta -b_i)}$, which is indeed the sub-case discussed by Ogasawara (2017, Theorem 5) and given by Equation (4) in Sect. 2. The transformations in Equation (52) are ability-dependent, and as such are automatically excluded if at least two individuals with different abilities are available and the mathematical form of the 4PL model is preserved. For the same rationale it must follow that all the $\ell ^i_B=0$ in System (45). As also $\ell =0$, then it follows that also all the $\ell ^i_F=0$ in System (45). Similar considerations can be done for System (51). Hence, under the assumption that the mathematical form of the 4PL model is preserved (i.e., the left-side added parameters must be constants), the trade-off between the 2PL and the left-side added parameters is solved in the 4PL by the same conditions of the 3PL model (i.e., two individuals with different abilities and two items with different discrimination). Since the identifiability conditions of the 2PL model are also the same, the only difference w.r.t. the 3PL model is in the minimal numbers of individuals and/or items required. In the fixed-effects 4PL model, the condition $N|Q|\ge n|Q|+N-I$ for $|Q|=2$, $n=4$, and $I=2$ yields $2N\ge 8+N-2$, that is $N\ge 6$, while in the random-effects 4PL model the condition $2^{|Q|}-1\ge n|Q|+2-I$ for $n=4$ and $I=2$ yields $2^{|Q|}\ge 4|Q|+1$, that is $|Q|\ge 5$.

This situation is not equivalent to the results of Ogasawara (2017, Proposition 2), which states that in addition to the conditions expressed by Wu (2016), either $c^*=c_i$ or $d^*_i=d_i$ must be set to identify the fixed-effects 4PL. Considering these conditions in the present perspective it appears that, although fixing either the $c_i$ or the $d_i$ parameters is sufficient to respectively set $\ell ^i_B=0$ in System (45) and $\ell ^i_F=0$ in System (51), these conditions are not necessary since preserving the form of the 4-parameter IRFs given at least two individuals with different abilities is a less demanding constraint that erases all empirically indistinguishable solutions, like the exclusive trade-off between the left-side added parameters captured by Equation (52). Nonetheless, if one takes into account the additional constraints imposed by Ogasawara (2017), which amounts to assume $I=2+|Q|$ (i.e, 2 conditions for the 2PL model and |*Q*| left-side added parameters fixed) into the condition $N|Q|\ge n|Q|+N-I$, one obtains for $|Q|=2$ exactly $N\ge 4$.

Two final remarks are in order for the 4PL case. The first one is that, similarly to the sub-case of Equation (52), the other sub-case introduced by Ogasawara (2017), and given by Equation (5) in Sect. 4.1, can be obtained from system (45). Let us first consider that, by system (45), it holds that

$$\begin{aligned} g_{q_i}(\theta ^*)=\frac{e^{a^*_i\theta ^*}}{e^{a^*_ib^*_i}+e^{a^*_i\theta ^*}} = \frac{e^{a_i\theta }+\ell }{e^{a_ib_i}+e^{a_i\theta }}= g_{q_i}(\theta ) + \frac{\ell }{e^{a_ib_i}+e^{a_i\theta }}. \end{aligned}$$

Let us then consider the solutions (28) obtained in Sect. 4.1 as a split of Equation (28). By replacing the KST quantities in the solutions (28) with the associated IRT quantities, one obtains the system

53 $$\begin{aligned} {\left\{ \begin{array}{ll} c_i-c^*_i = - (1- c^*_i)\frac{g_{q_i}(\theta )-g_{q_i}(\theta ^*)}{1-g_{q_i}(\theta )} = (1- c^*_i)\frac{\ell }{e^{a_ib_i}}\\ d_i-d^*_i = -d^*_i\frac{g_{q_i}(\theta )-g_{q_i}(\theta ^*)}{g_{q_i}(\theta )} = d^*_i\frac{\ell }{e^{a_i\theta }} \end{array}\right. } \quad \Leftrightarrow \quad {\left\{ \begin{array}{ll} c^*_i = \frac{ce^{a_ib_i}-\ell }{e^{a_ib_i}-\ell }\\ d^*_i = \frac{d_ie^{a_i\theta }}{e^{a_i\theta }+\ell } \end{array}\right. } \end{aligned}$$

where Equation (5) follows from the latter system.

The second remark is that the identifiability conditions for the 1PL-GS $P(X_i=1|\theta , \Gamma ^{-2}_i)$ can be obtained by assuming $a_i=1$ in transformations (45) and (51). Since now all discrimination parameters are equal, the rationale discussed for the 3PL and the 4PL cannot be applied anymore to set $\ell =0$. However, assuming at least two individuals with different abilities still constraints all empirically indistinguishable solutions, so that it must hold $\ell ^i_B=0$ for all items. It follows that $\ell ^i_F=\ell$ for all items so that the same condition of the 1PL-G can be used to identify the system, that is $c_1=c^*_1=0$. Similarly, the reversed system requires $d_1=d^*_1=0$. The condition $b_1=0$ sets the unidentifiability of the 1PL. Finally, the condition on the minimal numbers of individuals and/or items is given, in the fixed-effects 1PL-GS model, by the general condition $N|Q|\ge n|Q|+N-I$ that for $|Q|=2$, $n=3$, and $I=3$ yields $2N\ge 6+N-3$, that is $N\ge 3$, while in the random-effects 1PL-GS model is given by the condition $2^{|Q|}-1\ge n|Q|+2-I$ for $n=3$ and $I=3$ yields $2^{|Q|}\ge 3|Q|$, that is $|Q|\ge 4$.

## Discussion

In the present paper, it was suggested that the identification problems of IRT models for dichotomous items in the presence of left-side added parameters are related to the general issue of identifiability arising in knowledge structures in the presence of forward- or backward-gradedness w.r.t. an item. As the knowledge structure associated to the requirement of local stochastic independence in IRT is the power set, such structure is both BG and FG in all of the items, and as a result, every 4-parameter model presents a trade-off between the left-side added parameters and the remainder of the item response function (typically a 2-parameter model). This result has several consequences. First, this type of unidentifiability manifests itself as a trade-off between the left-side added parameters and the 2-parameter item response function and is therefore independent of the specific functional shape of the 2-parameter model, which could be a logistic as well as a normal ogive function or any other ogive model. Hence, application of the KST transformations to the IRT case allows to separate and distinguish between two different sources of unidentifiability: The first one concerns the trade-off between the left-side added parameters and the 2-parameter model, and the second one concerns the specific functional shape of the 2-parameter model. Sufficient and necessary conditions based on the KST transformations can be given to identify the former, while conditions are already known for the latter. As a consequence, IRT models appear to be identified when conditions for both sources have been given together with the needed minimal requirements on the number of individuals and/or items. As a result, conditions for the fixed effects 1PL-G and 3PL were recovered that match those already present in the literature, and conditions for the fixed effects 4PL were discussed. Similarly, conditions for the associated random-effects 1PL-G, 3PL, and 4PL models were discussed and appear to overlap those in the fixed-effects case while differing only w.r.t. the minimal conditions on the numbers of items. Most of all, it appears that the general beliefs on the identifiability of all these models are supported by the present work, although identifiability conditions need to be imposed to solve unidentified degenerate cases that arise from either equality in the discrimination parameters or in the abilities.

A second important consequence is that the KST transformations, once applied to the IRT context, can yield both empirically indistinguishable or unidentified solutions depending on how they are applied. Specifically, if they are applied ‘as they are,’ they yield empirically indistinguishable sets of IRFs that capture the trade-off described above without affecting the parameters within the 2-parameter IRF. If, instead, they are supplemented with an additional assumption that the mathematical forms of the 4- and/or 2-parameter must be preserved, then they present both empirically indistinguishable and unidentifiable solutions depending on which model is considered and on whether a fixed- or random-effects specification is considered. Generally, fixed-effects 4-parameter models are associated to empirical indistinguishability, while fixed-effects 3-parameter models are associated to unidentifiability. Random-effects models are always associated with empirical indistinguishability of the statistical model, even when the problem of the IRF is only one of unidentifiability, since the associated distribution of the ability is transformed. Most of all, the parameters within the 2-parameter IRFs become involved in the transformations. As a consequence, this might contribute to the occurrences in the literature of parameter instability that sometimes manifest themselves with different combinations of parameters as discussed in Sect. 2. Indeed, specific values of some parameters might bring the models close enough to some degenerate forms of them that are unidentifiable, thus creating instability in the parameter values. This might add up to the global identifiability issues of these models and to other estimation issues, like those occurring for items that are too easy or too hard, which are usually underrepresented in the outcomes.

Finally, since the IRT identifiability problem appears to be connected to the FG and BG nature of the power set structure, the results obtained might be beneficial for both KST and IRT models. Indeed, on the one hand, the fact that IRT models appear to be identifiable under suitable conditions, in spite of the fact that they are applied in the power set case, suggests that a KST-IRT perspective might provide a way to reduce the unidentifiability of knowledge structures. On the other hand, the use of structures that are known to be identified and are neither forward- nor backward-graded w.r.t. the items of interests might provide a substitute to the identifiability constraints that one needs to impose to solve the trade-off between the left-side added parameters and the IRFs. It is, however, important to stress that such an approach would imply abandoning the assumption of local stochastic independence in favor of applications of some generalized form of it, as suggested by Noventa et al. (2019), Noventa, Heller and Stefanutti (2021), and Ye, Kelava and Noventa (2023), or to work with SRFs in place of IRFs.

## A. Proof of KST Transformations (22)

In order to derive the transformations (22), we first derive the FG transformations (14), then the BG transformations (15), and then we combine them into the final result. Let us consider the transformations (14) for a FG structure. We are interested in the power set $2^Q$, which is FG in any *q*, and for which it holds for all items *q* that $\mathcal {K}_q = \overline{\mathcal {K}}^+_q$ so that $2^Q= \overline{\mathcal {K}}^+_q\cup \overline{\mathcal {K}}_q$. Transformations (14) take then the form

54 $$\begin{aligned} {\left\{ \begin{array}{ll} \beta '_q = \beta _q \\ \eta '_q = \eta _q+(1-\eta _q -\beta _q)(1-e^{t_F}) \\ \pi '(K) ={\left\{ \begin{array}{ll} \pi (K) + (1-e^{-t_F})\pi (K\setminus \{q\}) &{} \text {for all } K\in \mathcal {K}_q\\ e^{-t_F}\pi (K) &{} \text {for all } K\in \overline{\mathcal {K}}_q \end{array}\right. } \end{array}\right. } \end{aligned}$$

The last equation can be used to infer the shape for the transformations for the single item probabilities $g_q$ in the SLM. Indeed, we can obtain $g'_q$ as the marginal probability over all the states that contain *q*, namely

$$\begin{aligned} g'_q&= \sum _{K\in \mathcal {K}_q} \pi '(K) = \sum _{K\in \mathcal {K}_q} [ \pi (K)+(1-e^{-t_F})\pi (K\setminus \{q\})]\\&= \sum _{K\in \mathcal {K}_q} \pi (K)+ (1-e^{-t_F})\sum _{K\in \overline{\mathcal {K}}_q}\pi (K) = g_q+(1-e^{-t_F})(1-g_q) \end{aligned}$$

where the second passage follows from the fact that in the power set

$$\begin{aligned} \sum _{K\in \mathcal {K}_q}\pi (K\setminus \{q\})=\sum _{K\in \overline{\mathcal {K}}_q}\pi (K). \end{aligned}$$

For any other item $p\ne q$, one has instead that by construction, all states that contain an item *p* can be partitioned by the item *q* as $\mathcal {K}_p = (\mathcal {K}_p\cap \mathcal {K}_q)\cup (\mathcal {K}_p\cap \overline{\mathcal {K}}_q)$, which leads to

$$\begin{aligned} g'_p&= \sum _{K\in \mathcal {K}_p} \pi '(K) =\sum _{K\in \mathcal {K}_p\cap \mathcal {K}_q} \pi '(K)+\sum _{K\in \mathcal {K}_p\cap \overline{\mathcal {K}}_q} \pi '(K) \\&= \sum _{K\in \mathcal {K}_p\cap \mathcal {K}_q} [\pi (K)+(1-e^{-t_F})\pi (K\setminus \{q\})] +\sum _{K\in \mathcal {K}_p\cap \overline{\mathcal {K}}_q} e^{-t_F}\pi (K)\\&= \sum _{K\in \mathcal {K}_p\cap \mathcal {K}_q} \pi (K)+(1-e^{-t_F})\sum _{K\in \mathcal {K}_p\cap \overline{\mathcal {K}}_q}\pi (K) +\sum _{K\in \mathcal {K}_p\cap \overline{\mathcal {K}}_q} e^{-t_F}\pi (K)\\&= \sum _{K\in \mathcal {K}_p\cap \mathcal {K}_q} \pi (K)+\sum _{K\in \mathcal {K}_p\cap \overline{\mathcal {K}}_q} \pi (K) = \sum _{K\in \mathcal {K}_p} \pi (K) = g_p \end{aligned}$$

The resulting transformations w.r.t. item *q* for the set of parameters $\Gamma _\mathcal {K}=\{\beta ,\eta ,g\}$ are given by

55 $$\begin{aligned} {\left\{ \begin{array}{ll} \beta '_p = \beta _p &{}\text {for all } p\in Q\\ \eta '_p = \eta _p, \quad g'_p = g_p &{}\text {for all } p\ne q\\ \eta '_q = \eta _q+(1-\beta _q-\eta _q)(1-e^{t_F})&{}\\ g'_q = e^{-t_F}g_q +(1-e^{-t_F})&{} \end{array}\right. } \end{aligned}$$

Let us consider the transformations (15) for a BG structure. As before, we are interested in the power set structure $2^Q$, which is BG in any *q*, and for which it holds for all items *q* that $\mathcal {K}^-_q = \overline{\mathcal {K}}_q$ so that $2^Q=\mathcal {K}^-_q \cup \mathcal {K}_q$. Transformations (15) take then the form

56 $$\begin{aligned} {\left\{ \begin{array}{ll} \eta '_q = \eta _q\\ \beta '_q = \beta _q+(1-\eta _q -\beta _q)(1-e^{t_B})\\ \pi '(K) ={\left\{ \begin{array}{ll} \pi (K) + (1-e^{-t_B})\pi (K\cup \{q\}) &{} \text {for all } K\in \overline{\mathcal {K}}_q\\ e^{-t_B}\pi (K) &{} \text {for all } K\in \mathcal {K}_q \end{array}\right. } \end{array}\right. } \end{aligned}$$

As in the FG case, the last equation can be used to infer the shape for the transformations for the single item probabilities $g_q$. Indeed, $g'_q$ is the marginal probability over all the states that contain *q* that is

$$\begin{aligned} g'_q&= \sum _{K\in \mathcal {K}_q} \pi '(K) = \sum _{K\in \mathcal {K}_q} e^{-t_B}\pi (K) = e^{-t_B}g_q. \end{aligned}$$

For any other $p\ne q$ instead, one has that, as in the FG case, all states that contain an item *p* can be partitioned by the item *q* as $\mathcal {K}_p = (\mathcal {K}_p\cap \mathcal {K}_q)\cup (\mathcal {K}_p\cap \overline{\mathcal {K}}_q)$, which leads to

$$\begin{aligned} g'_p&= \sum _{K\in \mathcal {K}_p} \pi '(K) =\sum _{K\in \mathcal {K}_p\cap \mathcal {K}_q} \pi '(K)+\sum _{K\in \mathcal {K}_p\cap \overline{\mathcal {K}}_q} \pi '(K) \\&= \sum _{K\in \mathcal {K}_p\cap \mathcal {K}_q} e^{-t_B}\pi (K)+\sum _{K\in \mathcal {K}_p\cap \overline{\mathcal {K}}_q} [\pi (K)+(1-e^{-t_B})\pi (K\cup \{q\})]\\&= \sum _{K\in \mathcal {K}_p\cap \mathcal {K}_q} e^{-t_B}\pi (K)+\sum _{K\in \mathcal {K}_p\cap \overline{\mathcal {K}}_q} \pi (K)+\sum _{K\in \mathcal {K}_p\cap \mathcal {K}_q}(1-e^{-t_B})\pi (K)\\&= \sum _{K\in \mathcal {K}_p\cap \overline{\mathcal {K}}_q} \pi (K)+\sum _{K\in \mathcal {K}_p\cap \mathcal {K}_q}\pi (K) = g_p \end{aligned}$$

where the central passage follows from the fact that in the power set

$$\begin{aligned} \sum _{K\in \overline{\mathcal {K}}_q}\pi (K\cup \{q\})=\sum _{K\in \mathcal {K}_q}\pi (K). \end{aligned}$$

The resulting transformations w.r.t. item *q* for the set of parameters $\Gamma _\mathcal {K}=\{\beta ,\eta ,g\}$ are given by

57 $$\begin{aligned} {\left\{ \begin{array}{ll} \eta '_p = \eta _p &{}\text {for all } p\in Q\\ \beta '_p = \beta _p, \quad g'_p = g_p &{}\text {for all } p\ne q\\ \beta '_q = \beta _q+(1-\beta _q-\eta _q)(1-e^{t_B})&{}\\ g'_q = e^{-t_B}g_q &{} \end{array}\right. } \end{aligned}$$

The previously analyzed BG and FG cases are the main ingredients to build the full transformations. Indeed, the power set by nature is both BG and FG in all items of the domain, but as shown above, FG and BG affect only the parameters associated to a given item. As a last step, we can then derive the transformation associated to the $g_q$ parameters by imposing unidentifiability as a requirement, that is

$$\begin{aligned}&\eta _q+(1-\eta _q-\beta _q)g_q = \eta '_q+(1-\eta '_q-\beta '_q)g'_q\\&\eta _q+(1-\eta _q-\beta _q)g_q = \eta _q+(1-\beta _q-\eta _q)(1-e^{t_F})\\&\quad + [1-(\beta _q+(1-\beta _q-\eta _q)(1-e^{t_B}))-(\eta _q+(1-\beta _q-\eta _q)(1-e^{t_F}))]g'_q\\&(1-\eta _q-\beta _q)g_q = (1-\beta _q-\eta _q)(1-e^{t_F})+(1-\beta _q-\eta _q)[1-(1-e^{t_B})-(1-e^{t_F})]g'_q\\&g_q = (1-e^{t_F})+[1-(1-e^{t_B})-(1-e^{t_F})]g'_q, \end{aligned}$$

which yields the final result for the KST coefficient $g_q$

$$\begin{aligned} g'_q&= \frac{g_q-(1-e^{t_F})}{1-(1-e^{t_B})-(1-e^{t_F})} = \frac{g_q+e^{t_F}-1}{e^{t_B}+e^{t_F}-1}, \end{aligned}$$

and it is straightforward to verify that for $r_B=0$ or $r_F=0$ one retrieves exactly the transformations for the FG and BG cases. Finally, as to the ranges of $t_B$ and $t_F$, the condition $\eta '_q+\beta '_q <1$ implies $e^{t_F}+e^{t_B}>1$ since it holds that $(1-\eta _q-\beta _q)(2-e^{t_F}-e^{t_B})<1-\eta _q-\beta _q$, that is $1-e^{t_F}-e^{t_B}<0$. The conditions $\eta '_q, \beta '_q > 0$ respectively imply that $t_F<log{\frac{1-\beta _q}{1-\eta _q-\beta _q}}$ and $t_B<log{\frac{1-\eta _q}{1-\eta _q-\beta _q}}$. The condition $g'_q\in (0,1)$ implies that $1-e^{t_F}< g < e^{t_B}$ which can be split into the two conditions $t_{B} > \log {g_q}$ and $t_F > \log {(1-g_q)}$, which complete the results.
